# Human Management of a Wild Plant Modulates the Evolutionary Dynamics of a Gene Determining Recessive Resistance to Virus Infection

**DOI:** 10.1371/journal.pgen.1006214

**Published:** 2016-08-04

**Authors:** Nils Poulicard, Luis Fernández Pacios, Jean-Luc Gallois, Daniel Piñero, Fernando García-Arenal

**Affiliations:** 1 Centro de Biotecnología y Genómica de Plantas (UPM-INIA), and E.T.S.I. Agrónomos, Campus de Montegancedo, Universidad Politécnica de Madrid, Pozuelo de Alarcón, Madrid, Spain; 2 Centro de Biotecnología y Genómica de Plantas (UPM-INIA), Campus de Montegancedo, Pozuelo de Alarcón (Madrid) and Departamento de Sistemas y Recursos Naturales, E.T.S.I. Montes, Universidad Politécnica de Madrid (UPM), Madrid, Spain; 3 Institut National de Recherche Agronomique (INRA), UR1052, Génétique et Amélioration des Fruits et Légumes, Centre de Recherche PACA, Domaine Saint Maurice, CS60094, 84143, Montfavet, France; 4 Departamento de Ecología Evolutiva, Instituto de Ecología, Universidad Nacional Autónoma de México, México, D.F., México; John Innes Centre, UNITED KINGDOM

## Abstract

This work analyses the genetic variation and evolutionary patterns of recessive resistance loci involved in matching-allele (MA) host-pathogen interactions, focusing on the *pvr2* resistance gene to potyviruses of the wild pepper *Capsicum annuum glabriusculum* (chiltepin). Chiltepin grows in a variety of wild habitats in Mexico, and its cultivation in home gardens started about 25 years ago. Potyvirus infection of *Capsicum* plants requires the physical interaction of the viral VPg with the *pvr2* product, the translation initiation factor eIF4E1. Mutations impairing this interaction result in resistance, according to the MA model. The diversity of *pvr2/eIF4E1* in wild and cultivated chiltepin populations from six biogeographical provinces in Mexico was analysed in 109 full-length coding sequences from 97 plants. Eleven alleles were found, and their interaction with potyvirus VPg in yeast-two-hybrid assays, plus infection assays of plants, identified six resistance alleles. Mapping resistance mutations on a pvr2/eIF4E1 model structure showed that most were around the cap-binding pocket and strongly altered its surface electrostatic potential, suggesting resistance-associated costs due to functional constraints. The *pvr2/eIF4E1* phylogeny established that susceptibility was ancestral and resistance was derived. The spatial structure of *pvr2/eIF4E1* diversity differed from that of neutral markers, but no evidence of selection for resistance was found in wild populations. In contrast, the resistance alleles were much more frequent, and positive selection stronger, in cultivated chiltepin populations, where diversification of *pvr2/eIF4E1* was higher. This analysis of the genetic variation of a recessive resistance gene involved in MA host-pathogen interactions in populations of a wild plant show that evolutionary patterns differ according to the plant habitat, wild or cultivated. It also demonstrates that human management of the plant population has profound effects on the diversity and the evolution of the resistance gene, resulting in the selection of resistance alleles.

## Author Summary

Viruses cause plant diseases, whose severity is considered to increase under plant cultivation. Hence, it is highly relevant to understand the genetics of plant virus resistance, and its variation in wild and cultivated plants. Analyses of plant pathogen resistance have focused on R proteins, which recognize pathogen molecules triggering defenses according to a gene-for-gene interaction. Alternatively, infection may require the interaction of plant and pathogen molecules, mutations impairing this interaction resulting in recessive resistance according to a matching-alleles model. We analyse here the variation of a recessive resistance gene in wild and cultivated populations of a plant, focusing on chiltepin, a wild pepper currently undergoing incipient cultivation in Mexico. The *pvr2* gene encodes the translation initiation factor eIF4E1, which must interact with the viral VPg for potyvirus infection. A high genetic variation was found for *pvr2/eIF4E1* but, at odds with reports for *R* genes, there was no evidence for selection of resistance in wild chiltepin populations. However, data supported selection for resistance in cultivated populations, in spite of no phenotypic differences between wild and cultivated plants, and similar potyvirus incidences. Results demonstrate that cultivation has profound effects on the diversity and evolution of resistance.

## Introduction

Host-parasite interactions often show a high degree of genetic specificity, in that only a subset of parasite genotypes can infect and multiply in each host genotype [1–6]. The outcome (infection *vs*. resistance) of the host genotype-by-parasite genotype interaction can be integrated into coevolutionary models that differ in the underlying infection matrices [5]. The different proposed models stem from two general ones, the gene-for-gene (GFG) and the matching-alleles (MA) models, which were initially proposed to explain plant-parasite and invertebrate-parasite interactions, respectively [1,7], although evidence indicates that they are not taxonomically restricted [5]. These two models differ widely in their conceptual framework. In the GFG model, there is a hierarchy of resistance alleles in the host and infectivity alleles in the parasite, so that some host resistance alleles are intrinsically better than others, conferring resistance to a larger set of parasite genotypes, and similarly, some parasite alleles determining infectivity are intrinsically better than others, allowing infection of a larger set of host genotypes. In the MA model, there is no hierarchy of resistance (infectivity) alleles, and a particular host genotype is better at resisting a subset of parasite genotypes, and worse at resisting the rest of parasite genotypes, and a parasite genotype is better at infecting a subset of host genotypes, and worse at infecting the rest [1]. Both models also differ in the mechanisms determining host-parasite interactions. In the GFG model, infection occurs when the host genotype does not recognize the parasite genotype, i.e., matches between host and parasite molecules do not occur, while in the MA model successful infection requires molecular matches between host and parasite [5,7]. Hence, the evolution of resistance (infectivity) loci will differ if host-parasite interactions correspond to GFG or MA models. Notably, models predict that costs associated with resistance (infectivity) are required to maintain polymorphisms at resistance (infectivity) loci in the host (parasite) population in GFG interactions, but not in MA ones [1,7–9]. Accordingly, evidence of resistance costs has been reported for GFG interactions [10–12] but, to our knowledge, costs of resistance have not been analysed in MA interactions.

In the last 20 years a big progress has been made in understanding the molecular genetics of plant-parasite, including plant-virus, interactions. Resistance determined by single dominant genes (*R* genes) is based on host recognition of genotype-specific parasite molecules, being thus compatible with a GFG model, while recessive resistance prevents the matching of the specific host and parasite molecules required for infection, according to a MA model [13–17]. Molecular analyses of the genetic variation of resistance loci in host populations refer almost entirely to *R* genes determining resistance to cellular pathogens. *R* genes are considered to have evolved in response to the negative effects of parasite infection on the host fitness [13,18,19], that is, to virulence *sensu* [20]. Data from different systems show that *R* genes are hypermutagenic, and suggest that they are frequently under balancing selection [21]. In contrast with the effort devoted to understand the evolution of *R* genes, the molecular evolution of recessive resistance genes (in fact, susceptibility genes) has been seldom analysed. This gap is especially important in the case of plant-virus interactions, as a large fraction of monogenic resistance of plants to viruses is recessive [15,22]. Thus, the few published reports refer to plant-virus interactions [23–25], and focus on analyses of germplasm collections of crops, rather than on wild plant populations. Human-driven and natural selection on plant genomes can be very different in both cultivated and wild plant populations [26–29]. Thus, a full understanding of the evolutionary dynamics of MA-like plant-parasite interactions requires analyses in wild plant populations, as well as of comparisons between wild and cultivated ones.

Within this scenario, the aim of this work is to analyse the evolutionary patterns of plant recessive resistance loci involved in MA-like interactions, and how these patterns are affected by human management of the host populations. For this, we studied a wild plant that is currently undergoing incipient domestication, the wild pepper or chiltepin, *Capsicum annuum* var. *glabriusculum* (Dunal) [30]. Chiltepin is considered as the ancestor of the domesticated pepper *C*. *annuum* var. *annuum* L. [31], an economically important crop that was domesticated in Mesoamerica [32,33]. Chiltepin is a 5–10 year-lived perennial bush distributed from northern Colombia to south western United States. In Mexico, it grows in a variety of environments from the evergreen tropical forests of the Yucatan peninsula and the Gulf of Mexico to the dry deciduous forests of central and western Mexico and to the Sonoran desert [33–35]. Chiltepin plants grow and reproduce during the rainy season and their pungent fruits are consumed by birds, which disperse the seeds [34]. In some regions, fruits are harvested from wild populations for human usage [36] and their high value has led to its very recent cultivation. In the last 25 years, chiltepin cultivation has progressed from home gardens to monocultures in small traditional fields, where they are managed as an annual crop [35]. However, cultivated chiltepin does not show obvious phenotypic differences with wild populations and does not present any of the major traits of pepper domestication syndrome, such as larger, pendulous, non-deciduous fruits of different colours and pungency, flower morphology favoring selfing, and synchronized high germination rates [37]. Genetic variation is high in wild populations and shows a strong spatial structure associated with the biogeographical province of origin, and cultivation results in a significant loss of both genetic diversity and spatial genetic structure [35].

Wild and cultivated chiltepin populations are infected by potyviruses, reaching incidences of up to 42% according to population and year [38]. Thus, this work focuses on the recessive resistance gene *pvr2*, which has alleles in pepper (*Capsicum* spp.) conferring recessive resistance to virus species in the genus *Potyvirus* [39]. Potyviruses are a numerous group of economically important plant viruses with tubular particles encapsidating a single-stranded messenger-sense RNA genome of about 10000 nucleotides (nt), with a virus-encoded protein covalently linked to its 5’ end (VPg) and a polyadenylated tail at its 3’ end [40]. As for most characterized recessive resistance genes to viruses in plants [15,41,42], *pvr2* encodes an eukaryotic translation initiation factor, specifically, factor eIF4E1 [39]. Recessive resistance is expressed as immunity (no infection) or decreased virus multiplication [15,43,44], and the various *pvr2* resistance alleles reported differ from the susceptibility wild type allele in a small and mainly non-conservative number of amino acid changes [22,23,39,45]. It has been shown that the potyviral VPg interacts directly with pvr2/eIF4E1 in yeast two-hybrid and *in vitro* binding assays, and the physical interaction between pvr2/eIF4E1 and the virus VPg is required for virus infection [46–49], although the exact role in the potyvirus life cycle of eIF4E-VPg interaction remains a matter of discussion [15,50]. Mutations at pvr2/eIF4E1 that prevent its interaction with the VPg lead to resistance [22,23,51] and mutations at the VPg central domain that restore the pvr2/eIF4E1-VPg interaction allow infection [23]. Thus, the pvr2/eIF4E1-VPg-determined pepper-potyvirus interaction corresponds mechanistically to a MA model.

The *pvr2/eIF4E1* allelic diversity has been extensively screened in accessions of *C*.*annuum* var. *annuum* (domestic bell and chili pepper) and, to a lesser extent, in its relatives in the *Capsicum* genus, reporting one of the largest allelic series of eIF4E, including different susceptibility and resistance alleles to potyviruses [22,23,39,45,52,53]. Genetic variation and functional analyses have provided evidence of selection at *pvr2/eIF4E1* for potyvirus resistance [23]. However, these analyses were based for the largest part on accessions of domestic *Capsicum* species, and included few accessions of wild relatives, so that selection for potyvirus resistance could be associated with selection pressures (including potyvirus infection) specific of, or modulated by, the agroecosystem environment.

The reported incidence of potyviruses infection in chiltepin, together with the high genetic diversity of wild chiltepin populations in a variety of habitats in Mexico, and its incipient domestication, makes the chiltepin-potyvirus interaction a unique system to analyse the genetic variation and the evolutionary patterns of a recessive resistance gene (*pvr2/eIF4E1)*, as well as the potential effects of human management of a host plant and its habitat on the diversity and the evolution of resistance, the two goals of this study. To attain these goals we (i) obtained the nucleotide sequence of *pvr2/eIF4E1* in plants collected from wild and cultivated chiltepin populations in different biogeographical provinces of Mexico; (ii) analysed the genetic diversity and structure of *pvr2/eIF4E1* according the region of origin and the level of human management; (iii) identified and characterized functionally the different *pvr2/eIF4E1* alleles present in chiltepin populations; (iv) analysed the effect of these mutations on pvr2/eIF4E1 structure, (v) evaluated the frequency of potyvirus resistance in the populations and (vi) assessed the incidence of potyvirus infection in chiltepin populations. Our results suggest that resistance probably has associated costs due to functional constraints on pvr2/eIF4E1. Also, in wild chiltepin populations *pvr2/eIF4E1* accumulated synonymous changes, and the frequency of resistance alleles was low, while in cultivated populations *pvr2/eIF4E1* accumulated non-synonymous changes and the frequency of resistance alleles was significantly higher than in wild populations. These results are evidence of stronger selection for resistance under cultivation, and indicate a role of human management on the evolution of *pvr2/eIF4E1*.

## Results

### Diversity and genetic structure of the pvr2/eIF4E1 gene from chiltepin populations

The coding sequence of the *pvr2/eIF4E1* gene has a length of 687nt and encodes a predicted protein of 228 amino acids. The variability of the *pvr2/eIF4E1* coding sequence was evaluated in 97 chiltepin plants, 70 from wild and 27 from cultivated populations. These plants were randomly selected from 16 wild and 9 cultivated populations (2–4 plants per population) to represent the diversity of the species in six biogeographical provinces of Mexico (S1 Table). Note that neither the total number of sampled populations nor the ratio of wild to cultivated ones is evenly distributed across biogeographical provinces (S1 Table), which reflects the abundance of chiltepin and the intensity of cultivation [35]. A total of 12.4% of plants were identified as heterozygous at the *pvr2/eIF4E1* locus (S2 Table). The proportion of heterozygous plants was similar between wild and cultivated populations (*χ*^2^ = 1.3; *P* = 0.253), the same result being obtained when the plants from cultivated populations were compared with three random subsets of wild plants of the same size (*χ*^2^<30; *P*>0.083). For wild populations, the proportion of heterozygous plants significantly varied between biogeographical provinces (*χ*^2^ = 17.9; *P* = 0.003), which was due to the higher frequency of heterozygotes in AZP: when populations from this province were not included in the analysis, heterozygosity no longer depended on province (*χ*^2^ = 2.42; *P* = 0.659). From these 97 plants, a total of 109 coding sequences of the *pvr2/eIF4E1* gene were obtained, 77 from wild and 32 from cultivated populations, and 17 haplotypes were identified at the nucleotide sequence level (Table 1, S1 Table). No significant difference in haplotype richness was observed between wild and cultivated populations over all biogeographical provinces (*χ*^2^ = 2.4; *P* = 0.169) a result that, again, held regardless of sample size (*χ*^2^<1.5; *P* = 0.903).

**Table 1:** Number of analysed sequences and diversity of haplotypes of *pvr2/eIF4E1* coding sequence in chiltepin populations according to biogeographical province and habitats.

	*Number of sequences*			*Number of haplotypes*		
Region	W	C	Total	W	C	Total
**SON**	18	4	**22**	4	2	**4**
**CPA**	17	4	**21**	3	3	**6**
**AZP**	22	2	**24**	3	1	**3**
**SMO**	6	17	**23**	4	5	**8**
**CPS**	4	2	**6**	1	1	**2**
**YUC**	10	3	**13**	3	2	**4**
**Total**	**77**	**32**	**109**	**13**	**11**	**17**

Region indicates the biogegraphical province: Sonora (SON), Costa del Pacífico (CPA), Costa del Pacífico Sur (CPS), Altiplano Zacatecano-Potosino (AZP), Eastern side of the Sierra Madre Oriental (SMO), Yucatan (YUC). W = Populations from wild habitats, C = Cultivated populations.

The genetic diversity of the coding sequence was of 0.00359 ± 0.00115 nucleotide substitutions per site for the whole set of 109 *pvr2/eIF4E1* sequences and of 0.00655± 0.00130 for the concatenated sequenced introns (Table 2, see S3 Table for detailed intron diversity). Coding sequence diversity was highest in YUC and SMO, and lowest in SON and CPS (Table 2). Plants grown from seeds of fruits purchased at local markets were also analysed, named as local market populations. People selling the fruits claimed that they had been collected from local wild chiltepin populations, which was confirmed on the basis of the polymorphisms of nine microsatellite markers [35]. To further check if local market populations were derived from fruits harvested from wild populations and, thus, represented their genetic diversity, the genetic differentiation of the *pvr2/eIF4E1* coding sequences between wild and local market populations was analysed. The value of the fixation index *F*_*ST*_ between these two groups of populations was very low and not significantly different from zero (*F*_*ST*(W/LM)_<0.001, *P* = 0.388), showing no genetic differentiation between these two types of populations that, hence, can be clumped into a single class (wild populations). When the genetic diversity was analysed according to habitat, it was found to be 1.4 times higher in the cultivated than in the wild populations (0.00400 vs. 0.00292, Table 2) and the *F*_*ST*_ value between wild and cultivated populations (*F*_*ST*(habitat)_ = 0.208, *P*<0.001) indicated that *pvr2/eIF4E1* was genetically structured according to habitat, a result that held when the comparison was between sequences from cultivated plants and random subsets of sequences from wild plants of the same size (*χ*^2^>0.107; *P*<0.001).

**Table 2:** Genetic diversity of *pvr2/eIF4E1*.

			π ± SE		
	Exons, all	Exons, wild	Exons, cultivated	Introns, all	Exons + introns
**SON**	0.00098 ± 0.00070	0.00102 ± 0.00069	0.00098 ± 0.00092	0.00454 ± 0.00135	0.00339 ± 0.00098
**CPA**	0.00125 ± 0.00066	0.00050 ± 0.00039	0.00319 ± 0.00159	0.00714 ± 0.00128	0.00543 ± 0.00108
**AZP**	0.00110 ± 0.00080	0.00113 ± 0.00085	0.00000 ± 0.00000	0.00099 ± 0.00042	0.00089 ± 0.00039
**SMO**	0.00333 ± 0.00127	0.00492 ± 0.00187	0.00265 ± 0.00113	0.00020 ± 0.00014	0.00100 ± 0.00027
**CPS**	0.00079 ± 0.00077	0.00000 ± 0.00000	0.00000 ± 0.00000	0.00519 ± 0.00147	0.00381 ± 0.00108
**YUC**	0.00390 ± 0.00160	0.00265 ± 0.00137	0.00196 ± 0.00127	0.00140 ± 0.00077	0.00158 ± 0.00074
**overall**	0.00359 ± 0.00115	0.00292 ± 0.00106	0.00400 ± 0.00134	0.00655 ± 0.00130	0.00561 ± 0.00106
***d***_***S***_	0.00435 ± 0.00213	0.00504 ± 0.00262	0.00252 ± 0.00164	-	-
***d***_***N***_	0.00391 ± 0.00151	0.00305 ± 0.00145	0.00452 ± 0.00158	-	-
***d***_***N***_**/*d***_***S***_	0.89885	0.60516	1.79365	-	-

Genetic diversity (π) ± standard errors (SE) for exons or introns, for all populations (all) or only wild (wild) or cultivated (cultivated) populations. Data for concatenated exon plus intron sequences are presented for all populations. Data are presented according to biogeographical province as in Table 1, and for all provinces clumped together (overall). For the exons, the genetic diversity at synonymous (*d*_*S*_) and non-synonymous (*d*_*N*_) positions and the dN/dS ratios are also evaluated for all populations.

The diversity of the *pvr2/eIF4E1* coding sequences also showed a strong spatial structure, both at the population level (*F*_*ST*_ = 0.625, *P*<10^−4^ and *F*_*ST*_ = 0.643, *P*<10^−4^, for all or only wild populations, respectively) and at the level of the biogeographical province (*F*_*ST*_ = 0.522, *P*<10^−4^ and *F*_*ST*_ = 0.584, *P*<10^−4^, for all or only wild populations, respectively). More specifically, the chiltepin populations of each biogeographical province were genetically differentiated for the *pvr2/eIF4E1* coding sequences, except between CPS/SON, CPS/CPA and CPS/YUC regions (S4 Table). To analyse if this spatial structure followed a model of isolation by distance, a Mantel test was performed between the matrices of genetic and geographical distances among chiltepin wild populations. Data showed that the distribution of the genetic variation of *pvr2/eIF4E1* was not correlated with the geographic distance (*r* = 0.220, *P*>0.065; S1 Fig).

**Fig 1:**
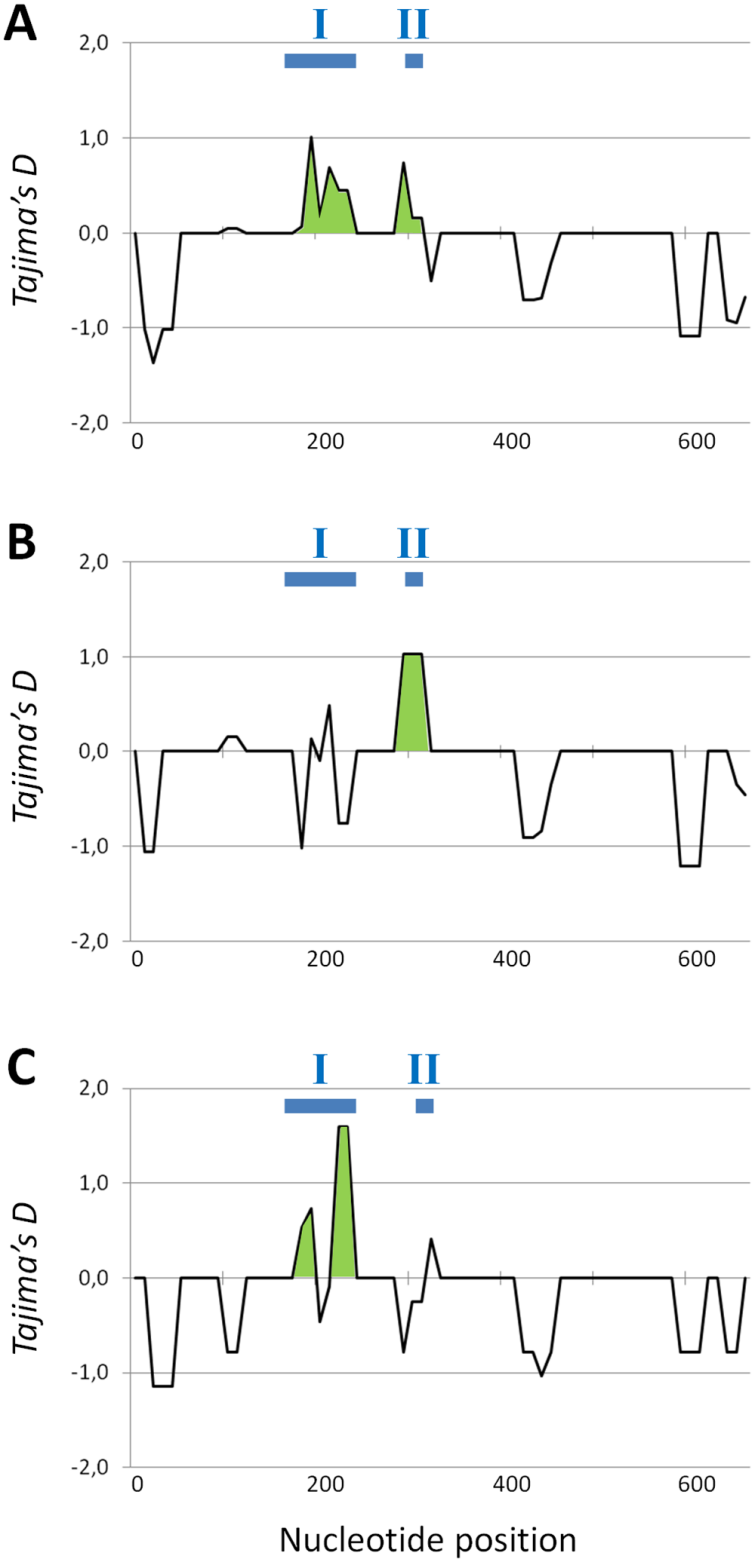
Tajima’s D (*D*_*T*_) values across the *pvr2/eIF4E1* coding sequences of all (A), wild (B) and cultivated (C) chiltepin populations. The green areas indicate the maximum of *D*_*T*_ values. I and II delimit the protein regions I and II involved in potyvirus resistance (as described in [23]).

Table 2 also shows the nucleotide diversity of the *pvr2/eIF4E1* coding sequence at synonymous and non-synonymous positions and the *d*_*N*_/*d*_*S*_ ratio indicates that *pvr2/eIF4E1* is globally under mild negative selection (*d*_*N*_/*d*_*S*_ = 0.899). When sequences from wild and cultivated populations were analysed separately, *d*_*N*_/*d*_*S*_ values were significantly different. Evidence for negative selection on *pvr2/eIF4E1* was stronger in wild populations (*d*_*N*_/*d*_*S*_ = 0.605), while it appeared to be under positive selection in cultivated populations (*d*_*N*_/*d*_*S*_ = 1.784). However, no site under positive selection was consistently identified by the different methods applied (see Material and Methods), either when all sequences were analysed together or according to habitat, wild or cultivated. Only codon 205 was identified as under positive selection by the REL method. Tajima’s *D* (*D*_*T*_) showed negative values for *pvr2/eIF4E1* (-0.691; -0.868 and -0.519 for all, wild and cultivated populations, respectively) which did not depart from the null hypothesis of neutrality. However, a sliding window analysis of *D*_*T*_ across the entire *pvr2/eIF4E1* coding sequence revealed regions with strongly positive *D*_*T*_ values, around codon 105 for wild populations and between codons 67 and 77 in cultivated populations (Fig 1). Positions 67–77 include those determining potyvirus resistance (see below) and position 105 has a polymorphism exclusive to AZP province.

### Identification of pvr2/eIF4E1 alleles in chiltepin populations

**Fig 2:**
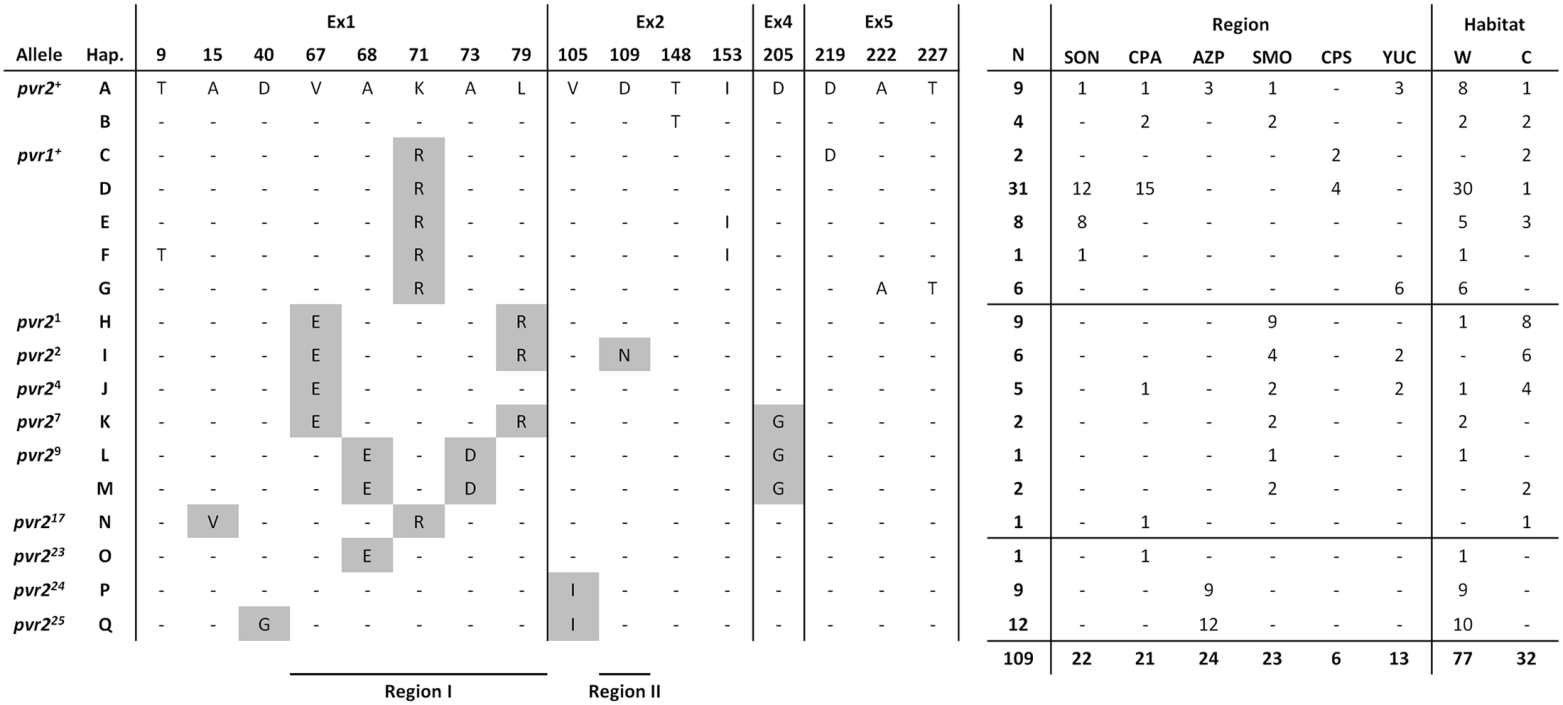
Amino acid polymorphisms in pvr2/eIF4E1 and *pvr2/eIF4E1* allele frequencies in chiltepin populations according to biogeographical province and habitat. Ex1 to Ex5 indicate the protein domains encoded by exons 1 to 5 of *pvr2/eIF4E1*, respectively. The different alleles and haplotypes at the nucleotide sequence level (Hap. A to Hap. Q) are indicated. N is the total number of sequences corresponding to each haplotype identified in chiltepin populations according to geographical provinces (SON: Sonora, CPA: Costa del Pacífico, CPS: Costa del Pacífico Sur, AZP: Altiplano Zacatecano-Potosino, SMO: Eastern side of the Sierra Madre Oriental, YUC: Yucatan) and habitats (W: wild, C: cultivated). Sequences are compared with that of *pvr2*^+^, “-”indicates that the codon is identical to that of *pvr2*^+^, the non highlighted letters identify codons where a synonymous substitution occurred, and the grey boxes highlight codons with non-synonymous substitutions. Regions I and II delimit the protein regions involved in Potyvirus resistance (as in [23]).

At the amino acid sequence level, a total of eleven allelic variants were identified based on 10 polymorphic sites, 7 of which were localized in exon 1 (Fig 2). Eight of these alleles had been reported previously within the *Capsicum* genus [22,23,45], three of them conferring susceptibility to potyviruses (*pvr2*^+^, *pvr1*^+^ and *pvr2*^17^) and five conferring resistance (*pvr2*^1^, *pvr*2^2^, *pvr2*^4^, *pvr2*^7^, *pvr2*^9^). The eight previously reported alleles represented 87 out of the 109 *pvr2/eIF4E1* sequences (i.e. 79.8%) obtained in this study (Fig 2). The 3 new alleles (named *pvr2*^23^ to *pvr2*^25^) were characterized by single (*pvr2*^23^ and *pvr2*^24^) or double (*pvr2*^25^) mutations relative to the reference allele *pvr2*^+^ (Fig 2). Interestingly, two of the three amino acid changes identified in these new alleles involved new polymorphic sites in comparison with previously reported alleles (codons 40 and 105, Fig 2). The three new alleles were identified in wild populations, allele *pvr2*^23^ was identified in CPA represented by only one sequence, and alleles *pvr2*^24^ and *pvr2*^25^ were identified in AZP, representing 21 out of the 24 sequences (87.5%) from this biogeographical province (Fig 2).

**Fig 3:**
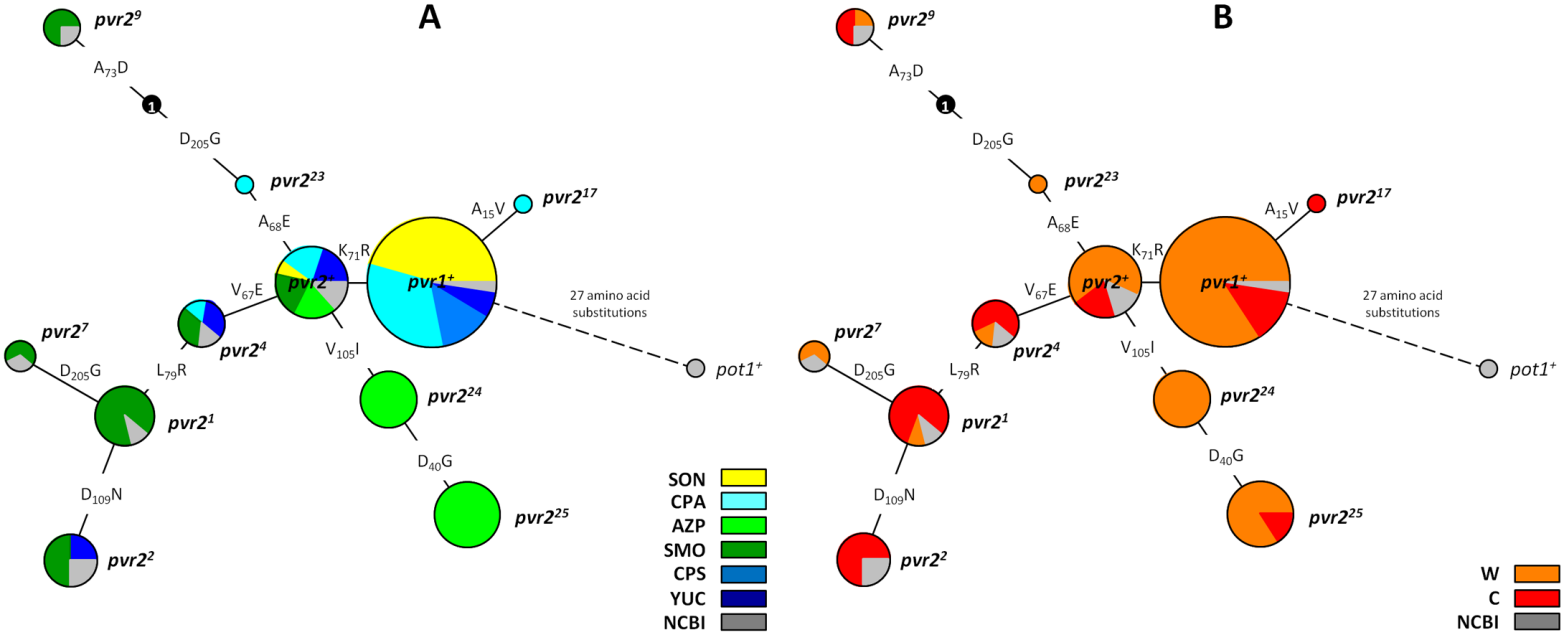
Minimum spanning network (MSN) of *pvr2/eIF4E1* coding sequences identified in chiltepin populations. Each *pvr2/eIF4E1* allele is indicated by one node in which the circle area is proportional to the number of individual sequences for this particular allele, and the biogeographical region (A) or the habitat (B) of origin of these sequences is represented as a proportional pie chart (in grey represents previously reported sequences available from the NCBI data base). The amino acid substitutions between alleles are indicated on the branches. One putative intermediate sequence connecting the *pvr2*^23^ and the *pvr2*^9^ alleles corresponds to the node labelled “1”.

A minimum spanning network (MSN) connecting all *pvr2/eIF4E1* alleles in the chiltepin population (Fig 3) showed that the tomato orthologous *pot-1*^+^/*eIF4E* used as outgroup was connected to the *pvr1*^+^ allele, which is the root of the network. The MSN also shows that most *pvr2/eIF4E1* alleles were connected by steps of just one amino acid substitution. Interestingly, the new allele *pvr2*^23^ corresponds to one of the most parsimonious putative intermediates described in Moury et al [44] to connect *pvr2*^+^ to *pvr2*^9^. However, one intermediate (labelled “1” in the network), needed to connect *pvr2*^23^ to *pvr2*^9^ is still missing, and sequence comparison of all previously described *pvr2/eIF4E1* alleles [22,23,45] did not reveal any sequence corresponding to this intermediate. MSN analysis demonstrated that the mutation D205G occurred at least twice in the evolution of *pvr2/eIF4E1* in chiltepin.

### Functional characterization of the new pvr2/eIF4E1 alleles identified and frequency of resistance in chiltepin populations

To test if the new *pvr2/eIF4E1* alleles identified in the chiltepin population were not impaired in the essential eIF4E1 function in mRNA translation, we analysed their ability to complement the eIF4E knockout yeast strain JO55 as in Charron et al [23]. Assays showed no growth difference in the selective medium between the yeasts complemented with the fully functional *pvr2/eIF4E1* susceptibility allele *pvr2*^*+*^ and the newly described ones (S2 Fig), strongly suggesting that alleles *pvr2*^*23*^, *pvr2*^*24*^ *and pvr2*^*25*^ are functional in translation.

**Fig 4:**
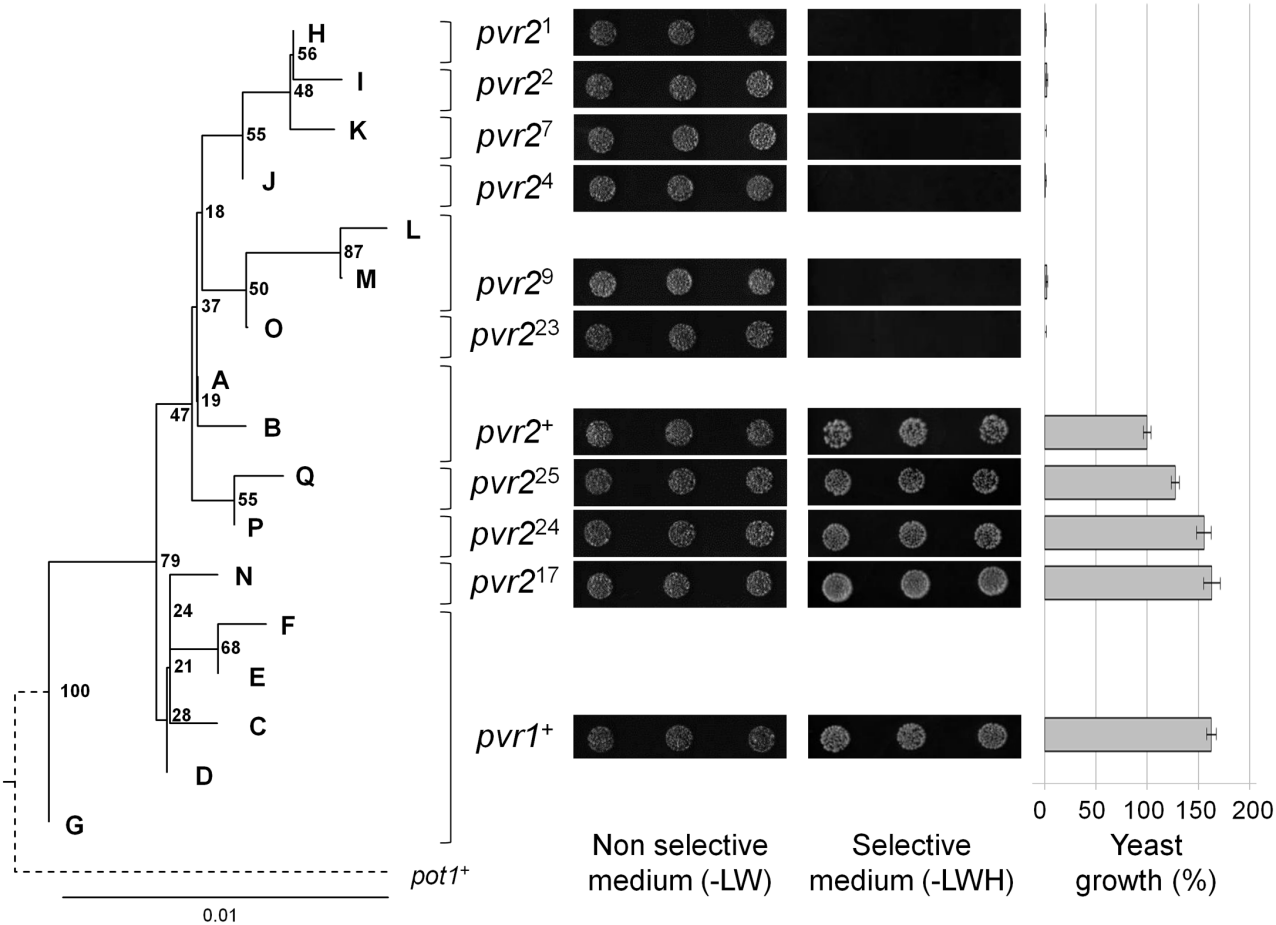
Phylogeny of *pvr2/eIF4E1* nucleotide sequence haplotypes and pvr2/eIF4E1-VPg:PVY interactions. The presented phylogeny was reconstructed by the NJ method using *pvr2/eIF4E1*, coding sequences. Bootstrap values (1000 replicates) are indicated on the nodes. A to Q are *pvr2/eIF4E1* haplotypes identified in chiltepin populations *pvr1*^+^ and *pvr2*^+^ to *pvr2*^25^ denote *pvr2/eIF4E1* alleles deduced from the *pvr2/eIF4E1* coding sequences; *pot1*^+^: *Potyvirus* susceptibility allele from tomato (*Solanum lycopersicum*, accession number AY723733). pvr2/eIF4E1-VPg:PVY interactions were evaluated by yeast two-hybrid assays. Yeast growth (%) indicates the percentage of the yeast growth on the selective medium (-LWH) compared to the reference yeast colonies co-transformed with pGADT7::*pvr2*^+^ and pGBKT7::VPg-PVY; standard errors were obtained after 3 replicates of the yeast two-hybrid assays in which 3 independent colonies for each pvr2/eIF4E1-VPg:PVY combination were randomly selected.

Next, for all the *pvr2/eIF4E1* alleles identified in chiltepin populations we analysed the interaction between eIF4E1 and viral VPg, as in the interaction of pepper with *Tobacco etch virus* (TEV) and *Potato virus Y* (PVY) there is strong correlation between absence of interaction and resistance. The physical interaction between the 11 pvr2/eIF4E1 proteins encoded and the VPg of the avirulent PVY-LYE84 isolate was analysed using yeast two-hybrid (Y2H) system. Differences of growth on selective medium were observed for yeast transformed with the constructs containing the different pvr2/eIF4E1 proteins and PVY-LEY84 VPg (Fig 4, S3 Fig), which confirmed the interaction pattern reported for the previously characterized alleles, i.e. interactions between the pvr2/eIF4E1-VPg for *pvr2*^+^ and *pvr1*^+^ susceptibility alleles, and no interaction for the resistance alleles *pvr2*^1^ to *pvr2*^9^. The proteins encoded by the *pvr2*^17^, *pvr2*^24^ and *pvr2*^25^-alleles interacted with the PVY-LYE84 VPg, suggesting that they are susceptibility alleles. In contrast, the eIF4E1 encoded by *pvr2*^23^ did not, suggesting it is a resistance allele toward PVY-LYE84 (Fig 4, S3 Fig). A detailed analysis of the effects of the mutations present in these alleles relative to *pvr2*^*+*^ (Fig 2), which has been taken as reference for susceptibility [23,44,45], showed that the single mutation V67E (characterising *pvr2*^4^) is sufficient to abolish the pvr2/eIF4E1-VPg interaction (S3 Fig). Similarly, the mutation A68E defining *pvr2*^23^ and also present in *pvr2*^9^, is sufficient to disrupt the pvr2/eIF4E1-VPg interaction (S3 Fig). Conversely, the single mutations A15V, D40G, K71R and V105I did not impair that interaction (S3 Fig). When these results were compared with a phylogeny of the *pvr2/eIF4E1* haplotypes, it was apparent that the interaction between pvr2/eIF4E1 and PVY-LYE84 VPg was more stable for the alleles corresponding to the most ancestral haplotypes (*pvr1*^+^, *pvr2*^+^, *pvr2*^17^, *pvr2*^24^ and *pvr2*^25^) than for the more derived *pvr2* alleles (*pvr2*^1^, *pvr2*^2^, *pvr2*^4^, *pvr2*^7^, *pvr2*^9^ and *pvr2*^23^ (Fig 4, see also Fig 3). Interaction assays were also performed between the *pvr2/eIF4E1* alleles identified in chiltepin populations and the VPg of TEV-HAT isolate, and demonstrated that the pvr2/eIF4E1-VPg interaction was efficient except for the *pvr2*^2^ allele as previously reported [23].

Finally, chiltepin plants were inoculated with isolates PVY-LYE84 and TEV-HAT (see Material and Methods) in order to confirm the susceptibility/resistance phenotypes of the new alleles deduced from the Y2H assays. Since the *pvr2*^17^ and *pvr2*^23^ alleles are infrequent in chiltepin populations (Fig 2), the CPA populations where they were found were not included in this analysis. However, as alleles *pvr2*^24^ and *pvr2*^25^ are prevalent in AZP (Fig 2), 40 plants from seeds of the BER-W population were inoculated with each virus, and all of them showed symptoms 21 days after inoculation and high viral accumulation as detected by ELISA. The *pvr2/eIF4E1* coding sequences were obtained from 10 randomly chosen plants among those inoculated with PVY-LYE84: 8 plants were homozygous for *pvr2*^24^, 1 plant was homozygous for *pvr2*^25^ and 1 plant was a *pvr2*^24^/*pvr2*^25^ heterozygote, which confirmed that the *pvr2*^24^ and *pvr2*^25^ alleles confer susceptibility to PVY-LYE84 and TEV-HAT.

Altogether, the described assays indicated that 6 out of 11 pvr2/eIF4E1 alleles found in chiltepin populations confer resistance to PVY-LYE84 infection. However, most *pvr2/eIF4E1* sequences obtained in this study (83 out of 109, i.e. 76.1%) correspond to susceptibility alleles. When the distribution of resistance alleles in the sampled plants was analysed, it was found that 20.6% of plants would be resistant to PVY-LYE84 (Table 3). Resistance frequency significantly differed among biogeographical provinces (for all populations: *χ*^2^ = 58.2, *P*<10^−4^; for wild populations: *χ*^2^ = 29.5, *P*<10^−4^; for cultivated populations: *χ*^2^ = 20.2, *P* = 10^−4^), being highest in populations from SMO and YUC (for overall population: 84.2% and 25.0%, respectively; for wild populations: 66.7% and 10.0%, respectively; for cultivated populations: 92.3% and 100.0%, respectively; Table 3). Interestingly, the frequency of resistant plants was significantly higher in cultivated populations than in wild ones (55.6% and 8.6%, respectively, *χ*^2^ = 25.4; *P*<10^−4^; S5 Table, Table 3).

**Table 3:** Frequency of potyvirus resistance alleles and resistant plants according to biogeographical province and habitat.

	N_Seq_ total	N_Seq_ S	N_Seq_ R	% R alleles	N_plants_ total	N_plants_ S	N_plants_ R	% R plants
**All**								
**SON**	22	22	0	0.0	22	22	0	0.0
**CPA**	21	19	2	9.5	20	18	2	10.0
**AZP**	24	24	0	0.0	18	18	0	0.0
**SMO**	23	3	20	87.0	19	3	16	84.2
**CPS**	6	6	0	0.0	6	6	0	0.0
**YUC**	13	9	4	30.8	12	9	3	25.0
**W**	77	71	6	7.8	70	64	6	8.6
**C**	32	12	20	62.5	27	12	15	55.6
**Total**	**109**	**83**	**26**	**23.9**	**97**	**77**	**21**	**21.6**
**Wild**								
**SON**	18	18	0	0.0	18	18	0	0.0
**CPA**	17	16	1	5.9	16	15	1	6.3
**AZP**	22	22	0	0.0	16	16	0	0.0
**SMO**	6	2	4	66.7	6	2	4	66.7
**CPS**	4	4	0	0.0	4	4	0	0.0
**YUC**	10	9	1	10.0	10	9	1	10.0
**Total**	**77**	**71**	**6**	**7.8**	**70**	**64**	**6**	**8.6**
**Cultivated**								
**SON**	4	4	0	0.0	4	4	0	0.0
**CPA**	4	3	1	25.0	4	3	1	25.0
**AZP**	2	2	0	0.0	2	2	0	0.0
**SMO**	17	1	16	94.1	13	1	12	92.3
**CPS**	2	2	0	0.0	2	2	0	0.0
**YUC**	3	0	3	100.0	2	0	2	100.0
**Total**	**32**	**12**	**20**	**62.5**	**27**	**12**	**15**	**55.6**

Frequency of potyvirus resistance in all wild and cultivated populations. Number of *pvr2/eIF4E1* coding sequences encoding alleles of susceptibility or resistance to potyviruses (N_Seq_), or number of plants predicted to be susceptible or resistant to potyviruses (N_plants_) according to the analyses of their *pvr2/eIF4E1* coding sequences, the results of yeast two-hybrid assays and/or potyvirus inoculations. Susceptibility (S); Resistance (R); % R alleles: percentage of resistance alleles; % R plants: percentage of resistant plants.

### Effects of pvr2/eIF4E1 mutations on the protein structure

Most previously reported mutations in the pvr2/eIF4E1 protein of *Capsicum* spp. resulting in potyvirus resistance were predicted to be in the cap binding pocket [23,54]. None of the amino acid substitutions detected in pvr2/eIF4E1 of chiltepin relative to *pvr2*^+^, except D109N, were located at the sites interacting with the mRNA m7GTP cap or the eIF4G factor (S4 Fig). Since no experimental structure is available for the eIF4E1 protein of *Capsicum*, a three-dimensional model was built in order to locate and to predict the structural effects in pvr2/eIF4E1 of the mutations identified in chiltepin. First, the amino acid sequence of the *C*. *annuum* var. *annuum pvr2*^+^ reference allele was aligned with those of eIF4E proteins with known crystal structure (from *Homo sapiens*, *Mus musculus*, *Triticum aestivum*, and *Pisum sativum*). A phylogeny of these five eIF4E was reconstructed (S5A Fig), and their secondary structures were compared (S5B Fig), which showed a very high conservation except for the N-terminal domain which is longer in human, wheat and pepper (S5B Fig). The non-conserved N-terminal domain was demonstrated to be flexible in yeast [55], and our analysis confirmed that this domain is predicted to be disordered in human, wheat and in *Capsicum* (S5B and S5C Fig).

**Fig 5:**
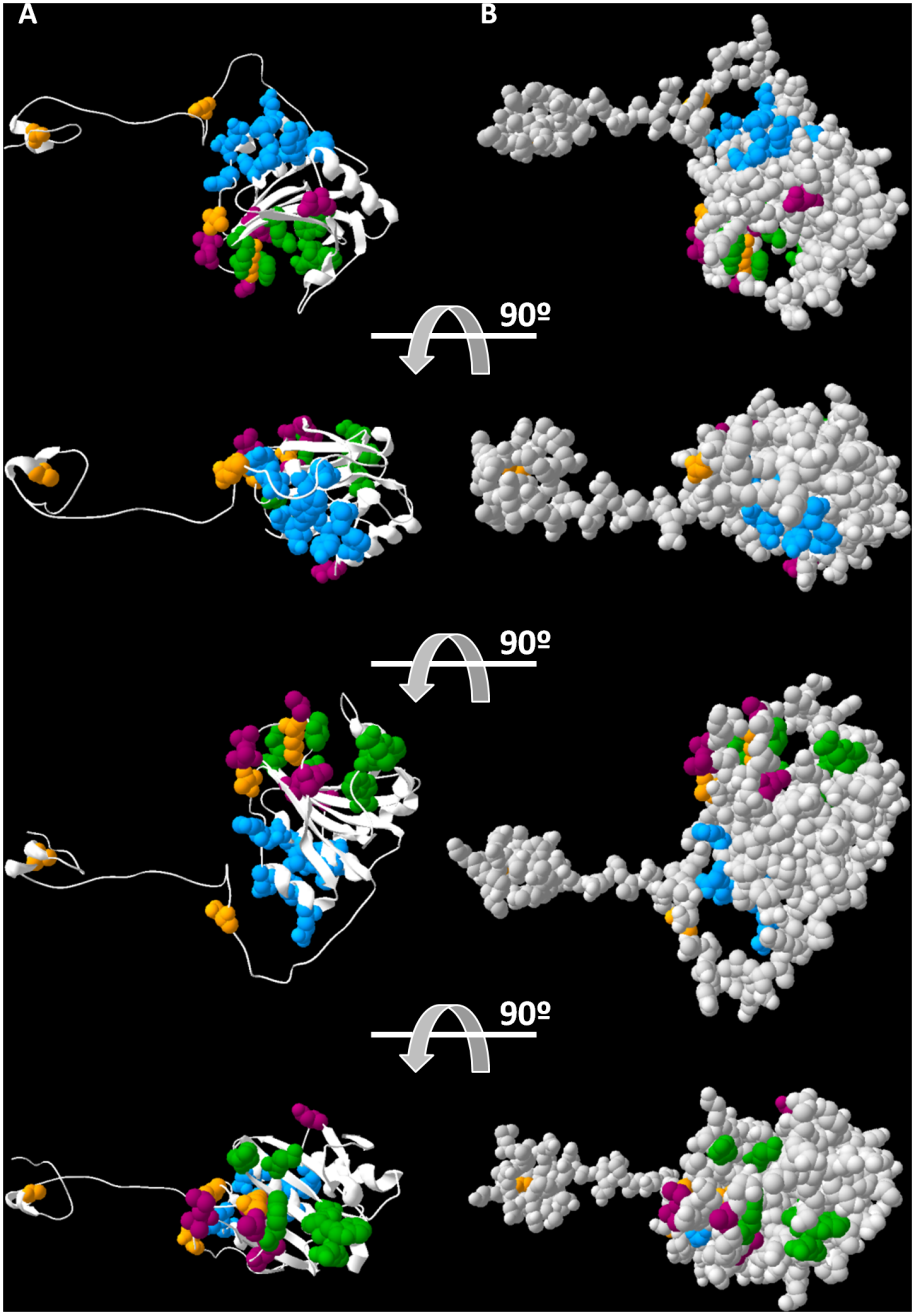
3D-model of the pvr2/eIF4E1 protein structure. The localisation of the polymorphic positions identified in chiltepin populations (orange), sites involved in potyvirus resistance (purple), sites involved in the m7GTP cap recognition (green) and the domain of eIF4G interaction (blue) are indicated. (A) 3D-model representing the secondary structure of pvr2/eIF4E1 protein. (B) 3D-model representing the Van der Waals surface of pvr2/eIF4E1 protein.

The 3D-models generated independently for the 11 pvr2/eIF4E1 alleles in Fig 2 confirmed, first, that the N-terminal domain of pvr2/eIF4E1 is flexible, and second, that the structural core of pvr2/eIF4E1 protein is not significantly altered by any amino acid substitution identified in chiltepin populations (S6 Fig). With the single exception of residue 109, which is placed in the β strand spanning amino acid positions 107–115, all the analyzed mutations involve residues located at loops (S6 Fig). Loops connecting secondary structure elements exhibit a great conformational flexibility and are usually exposed to the aqueous environment. Correspondingly, all mutations in pvr2/eIF4E1 alleles locate at the protein surface and, interestingly, they are close to the domain involved in the m7GTP cap recognition and far distant from the interface associated with eIF4G recruitment (Fig 5). It must be also noticed that being part of the disordered N-terminal region, the mutation A15V and to a lesser extent, the mutation D40G, should not alter significantly the essential functions of the pvr2/eIF4E1 protein.

**Fig 6:**
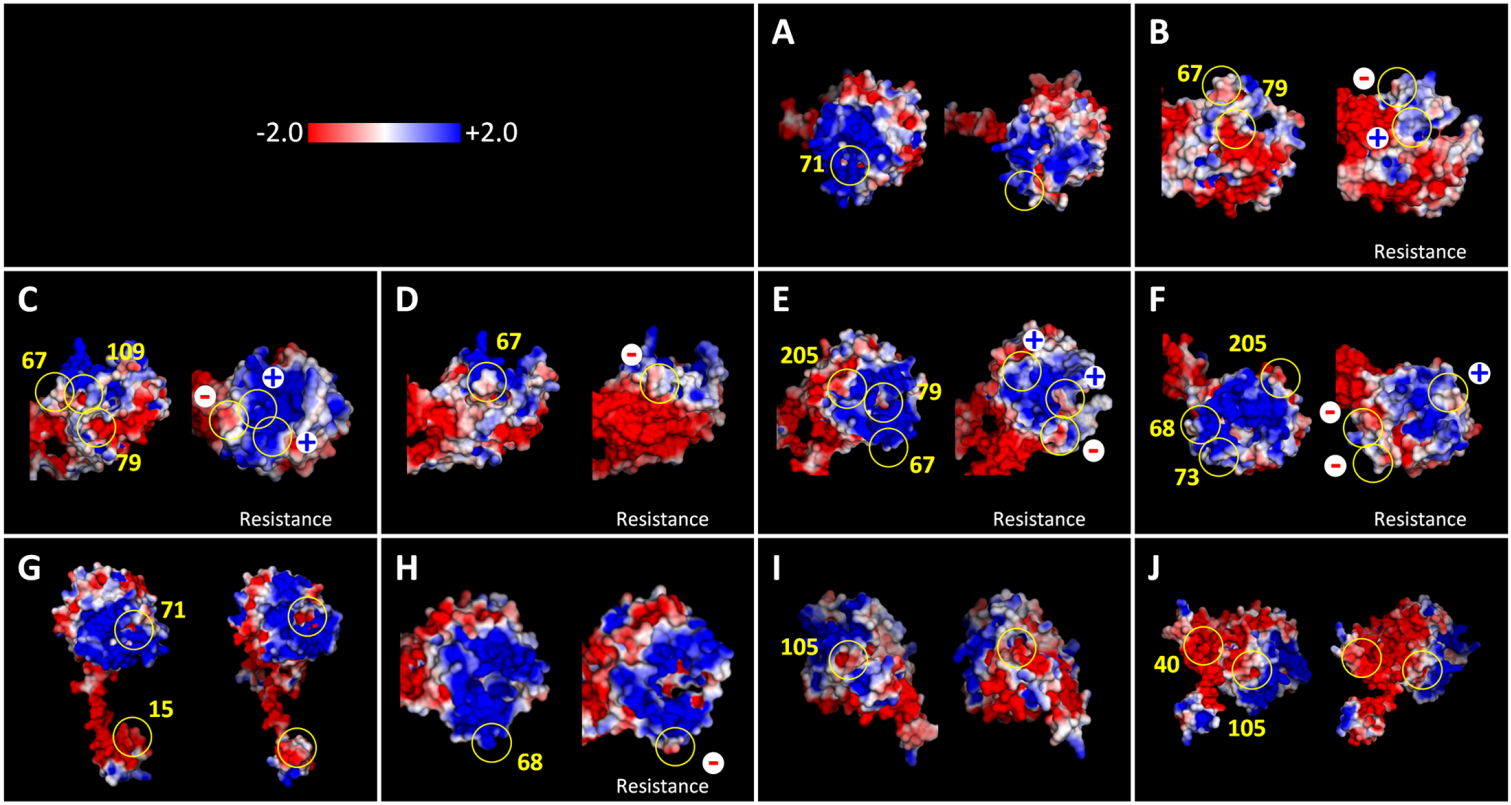
Poisson-Boltzmann electrostatic potential mapped onto the Van der Waals surfaces of pvr2/eIF4E1 protein. The electrostatic potential of the protein encoded by the reference allele *pvr2*^+^(left) is compared with that of alleles (right) *pvr1*^+^ (A), *pvr2*^1^ (B) *pvr2*^2^ (C), *pvr2*^4^ (D), *pvr2*^7^ (E), *pvr2*^9^ (F), *pvr2*^17^ (G), *pvr2*^23^ (H), *pvr2*^24^ (I) and *pvr2*^25^ (J). Panels A, G, I and J compare the protein encoded by *pvr2*^+^ with those encoded by potyvirus susceptibility alleles, while panes B-F and H compare the protein encoded by *pvr2*^+^ with those encoded by potyvirus resistance alleles.

In addition to being localized at the surface of the protein (Fig 5), most amino acid substitutions (6 out of 10) involved steric changes associated to side chain volumes (except for A15V, K71R, V105I and D109N mutations) as well as noticeable local variations of the electrostatic potential in the protein surface (Fig 6). For the new alleles *pvr2*^23^, *pvr2*^24^ and *pvr2*^25^, only the mutation A68E in *pvr2*^23^ introduced a large change in electrostatic potential relative to *pvr2*^+^, from a strong positive to a clearly negative potential in the external surface of the protein (Fig 6). It is interesting to note that there is a perfect correlation between all significant changes of electrostatic potential in pvr2/eIF4E1 and the disruption of its interaction with PVY VPg (Fig 6). Our results reveal that drastic changes in the local electrostatic potential of surface regions caused by some mutations (e.g. neutral to negative in V67E or neutral to positive in L79R) have a great impact in terms of disrupting the interaction with PVY-LYE84 VPg. Finally, as the N-terminal tails are disordered in the 3D models of all 11 pvr2/eIF4E1 alleles, variations among alleles in the electrostatic potential of those disordered regions are in part translated to nearby regions of the structural core. This is why the electrostatic potential of the structurally conserved core is not exactly the same in all alleles, which could indirectly alter the function of the pvr2/eIF4E1 protein.

### Potyvirus incidence in chiltepin populations

To estimate the incidence of potyvirus infection in chiltepin populations, we analysed by ELISA leaf samples of 955 plants collected in wild and cultivated populations between 2007 and 2010. A total of 147 samples were ELISA positive, indicating a global *Potyvirus* incidence of 15.4% (Table 4). Potyvirus incidence varied significantly according to biogeographical province (*χ*^2^ = 50.2, *P*<10^−4^), being highest in SON and AZP (23.8% and 24.1%, respectively), where *pvr2/eIF4E1* resistance alleles were not identified. Potyvirus incidence varied significantly according to year (from 8.5% in 2008 to 22.2% in 2010; *χ*^2^ = 15.0, *P* = 0.002). This temporal variation was solely due to wild populations, in which incidence varied according to year (*χ*^2^ = 24.1, *P*<10^−4^; Table 4), which was not the case for the cultivated ones (*χ*^2^ = 1.5, *P* = 0.676; Table 4), indicating a more constant challenge of virus infection in human-managed populations. Habitat, wild or cultivated, was not a factor on *Potyvirus* incidence (*χ*^2^ = 0.3, *P* = 0.597; Table 4), however, the percentage of infected plants that showed disease symptoms (mosaic, leaf distortion) was significantly higher in cultivated than in wild populations (45.5% and 9.8%, respectively; *χ*^2^ = 24.6, *P*<10^−4^) whereas it did not differ according to biogeographical province (*χ*^2^ = 7.3, *P* = 0.202) (Table 5).

**Table 4:** *Potyvirus* incidence according to year of sampling, biogeographical province and habitat.

	All				Wild				Cultivated			
	N total	N (-)	N (+)	Incidence ± SE	Ntotal	N (-)	N (+)	Incidence ± SE	N total	N (-)	N (+)	Incidence ± SE
**2007**	260	229	31	11.9 ± 3.1	193	175	18	9.3 ± 10.4	67	54	13	19.4 ± 15.3
**2008**	165	151	14	8.5 ± 2.6	116	110	6	5.2 ± 2.9	66	58	8	12.1 ± 9.5
**2009**	467	379	88	18.8 ± 4.2	262	208	54	20.6 ± 6.2	205	171	34	16.6 ± 19.9
**2010**	63	49	14	22.2 ± 8.7	54	41	13	24.1 ± 11.4	1	1	0	0.0 ± 0.0
**SON**	164	125	39	23.8 ± 8.7	91	78	13	14.3 ± 8.3	73	47	26	35.6 ± 22.3
**CPA**	205	192	13	6.3 ± 4.1	135	131	4	3.0 ± 1.1	70	61	9	12.9 ± 20.2
**AZP**	249	189	60	24.1 ± 4.8	235	179	56	23.8 ± 5.1	14	10	4	28.6 ± 25.7
**SMO**	107	94	13	12.1 ± 10.4	9	2	7	77.8 ± 0.0	98	92	6	6.1 ± 5.1
**CPS**	128	123	5	3.9 ± 1.7	88	84	4	4.5 ± 2.6	40	39	1	2.5 ± 3.8
**YUC**	102	85	17	16.7 ± 8.5	58	50	8	13.8 ± 15.7	44	35	9	20.5 ± 3.0
**W**	616	524	92	14.9 ± 3.9								
**C**	339	284	55	16.2 ± 3.6								
**Total**	**955**	**808**	**147**	**15.4 ± 2.7**	**616**	**524**	**92**	**14.9 ± 3.9**	**339**	**284**	**55**	**16.2 ± 16.7**

Potyvirus incidence in all, wild and cultivated populations. N total: total number of samples analysed; N(-): number of samples negative for potyvirus detection, N(+): number of samples positive for potyvirus detection; Incidence values and standard errors (SE) are indicated as percentage of plants positive for potyvirus detection.

**Table 5:** Frequency of disease symptoms in infected plants according to year of sampling, geographical province and habitat.

	N_infected_	N_symptomatic_	N_asymptomatic_	% symptomatic
**2007**	31	1	30	3.2
**2008**	14	6	8	42.9
**2009**	88	25	63	28.4
**2010**	14	2	12	14.3
**SON**	39	10	29	25.6
**CPA**	13	4	9	30.8
**AZP**	60	12	48	20.0
**SMO**	13	1	12	7.7
**CPS**	5	0	5	0.0
**YUC**	17	7	10	41.2
**W**	92	9	83	9.8
**C**	55	25	30	45.5
**Total**	**147**	**34**	**113**	**23.1**

Disease symptoms: mosaic and leaf distortion. N_infected_: number of infected plant detected by DAS-ELISA; N_symptomatic_ and N_asymptomatic_: number of symptomatic and asymptomatic plants, respectively; W: wild populations; C: cultivated populations.

To identify which *Potyvirus* species infected chiltepin populations in Mexico, we amplified a highly conserved region of NIb gene from the most ELISA positive samples. Amplification was successful from 8 samples, 4 from AZP, collected in 2008 and 2009, 3 from SON, 2007, and 1 from CPA, 2009, yielding two groups of sequences: those from SON and CPA were 99% identical to *Pepper mottle virus* (PepMoV), and those from AZP were 83% identical to *Tobacco etch virus* (TEV) (S7 Fig). Amplification and sequence determination of the genes encoding the VPg and CP in these samples confirmed the results based on the NIb fragment (VPg: 97% and 77% of identity with PepMoV and TEV, respectively; CP: 98% and 83% of identity with PepMoV and TEV, respectively). The TEV-like potyvirus differed in 30 out of the 188 VPg amino acid positions from TEV but none of them included a site reported to be involved in *pvr2* resistance-breaking (S8 Fig).

## Discussion

In this study, the genetic diversity of the recessive resistance gene *pvr2/eIF4E1* to potyviruses was analysed in the wild ancestor of domesticated pepper, *Capsicum annuum* var. *glabriusculum* (chiltepin), with the aim of inferring the evolutionary pattern of a resistance locus involved in matching-allele (MA)-like interactions, and of evaluating the impact of incipient domestication on that pattern. For that, we compared the diversity of *pvr2/eIF4E1* for wild and cultivated chiltepin populations in six biogeographic provinces within its distribution range in Mexico, and we determined the phenotype of susceptibility or resistance of *pvr2/eIF4E1* alleles by the analysis of the interaction between pvr2/eIF4E1 and PVY-LYE84 VPg in a yeast two hybrid (Y2H) assay, and by the response of plants to viral inoculations. Infection requires the physical interaction between pvr2/eIF4E1 and the potyviral VPg, and it has been shown that there is a perfect correlation between pvr2/eIF4E1-VPg interaction-no interaction in Y2H and susceptibility-resistance in plants [22,23,51]. Also, the lack of physical interaction between pvr2/eIF4E1 and PVY-LYE84 VPg has been shown to be an efficient way of identifying resistance to potyviruses in *Capsicum* spp. However, interactions of particular pvr2/eIF4E1 resistance alleles with the VPg of other potyviruses may be more stable, resulting in susceptibility. Indeed, among the 25 previously described pvr2/eIF4E1 alleles, 23 confer resistance to PVY-LYE84 and only one to TEV-HAT [22,23,44,56].

In 109 *pvr2/eIF4E1* full-length coding sequences obtained from 97 chiltepin plants, 17 haplotypes were identified at the nucleotide sequence level, which largely differed in frequency. The most frequent one, haplotype D, accounted for 28% of total sequences, and the other four haplotypes encoding the susceptibility allele *pvr1*^*+*^, which according to the minimum spanning network (MSN) and phylogenetic analyses represents the basal state of *pvr2/eIF4E1* in chiltepin (Figs 2 and 3), accounted for 44% of total sequences (Fig 2). Allele frequency also varied according to biogeographical province, so that the genetic diversity of *pvr2/eIF4E1* coding sequence was 2.5–5 times higher in YUC and SMO than in the other four biogeographical provinces (Table 2). Also, the most basal *pvr2/eIF4E1* haplotype (G, Fig 2) was only identified in YUC. These results are consistent with the higher genetic diversity of chiltepin in YUC and SMO estimated from nuclear microsatellite makers (SSRs) [35] and with reports that identify the Yucatan peninsula and the areas around the Gulf of Mexico as centres of diversity and domestication of *C*. *annuum* [33,57]. Analyses of nuclear SSRs have shown a strong spatial structure of chiltepin genetic diversity according to biogeographical province [35], which was also the case for *pvr2/eIF4E1*, both when the coding sequence or the introns (S3 Table) were analysed. However, at odds with results from SSRs, which showed evidence of isolation by distance, the genetic distance among chiltepin populations at *pvr2/eIF4E1* poorly correlated with geographical distance. The discrepancy between the spatial structure of the variation of putatively neutral genetic markers and of *pvr2/eIF4E1* suggests that this gene is under selection associated with environment-specific factors. Although other factors may certainly be involved, selection on *pvr2/eIF4E1* could be associated with resistance to potyviruses, as potyvirus incidence differs according to biogeographical province (Table 4).

In agreement with the hypothesis that there is selection on *pvr2/eIF4E1* for resistance, MSN and phylogenetic analyses indicate that *pvr2/eIF4E1* has evolved to confer potyvirus resistance. Most *pvr2/eIF4E1* alleles can be connected by just one amino acid substitution, and the allelic diversity found in chiltepin allowed to identify alleles, as *pvr2*^23^, which were predicted as most parsimonious intermediates in pvr2/eIF4E1 evolution by Moury et al [44] (Fig 3). Analyses showed that the susceptibility allele *pvr1*^*+*^ is at the base of *pvr2/eIF4E1* phylogeny. From that state, evolution has proceeded towards decreasing the stability of the interaction between pvr2/eIF4E1 and PVY-LYE84 VPg, i.e., towards resistance, as judged by yeast growth in a selective medium complemented by a Y2H assay interaction (Fig 4). The most supported node in *pvr2/eIF4E1* phylogeny splits haplotypes encoding susceptibility alleles *pvr1*^*+*^ and *pvr2*^17^, from a cluster built of two less strongly supported subclusters, one including haplotypes corresponding to susceptibility alleles *pvr2*^*24*^ and *pvr2*^*25*^, and the other including haplotypes corresponding to susceptibility alleles *pvr2*^*+*^, from which all other haplotypes, encoding resistance alleles, derive (Fig 4). The pattern of evolution into this last cluster including both susceptibility and resistance alleles is compatible with a hypothesis of selection on *pvr2/eIF4E1* resulting in the evolution of a variety of resistance alleles, as was concluded from the analysis of a set of 25 accessions of *Capsicum annuum* [23]. Interestingly, when the phylogeny of all reported *pvr2/eIF4E1* alleles was reconstructed, resistance also appeared as a derived state, and evolution to resistance occurred in different phylogenetic clusters (S9 Fig). Although support for the internal nodes of the phylogeny was not strong, the topology was consistent regardless of the method of phylogenetic reconstruction, or when the phylogeny was based on only first and second codon positions (S9 and S10 Figs). Phylogenies derived from third codon positions (S11 Fig) did not present an informative pattern, supporting the significance of the main clusters in the other phylogenies. However, at odds with previous analyses [23], when the alleles in our chiltepin data set are considered, evidence of selection for resistance is weaker: most (10/17) haplotypes encoded susceptibility alleles and a large number of *pvr2/eIF4E1* polymorphisms in the chiltepin population were due to synonymous nucleotide substitutions, so that 7/17 haplotypes encoded the susceptibility alleles *pvr1*^*+*^ (5 haplotypes) and *pvr2*^*+*^ (2 haplotypes). In contrast, only non-synonymous mutations were found in the data set analysed by Charron et al [23]. Accordingly, no site, including those that determine potyvirus resistance, was identified in our data set as being under positive selection, with the possible exception of codon 205, in which the mutation D205G confers potyvirus resistance and occurred at least twice during *pvr2/eIF4E1* evolution in chiltepin (Fig 3). Positive selection on codons involved in potyvirus resistance was only detected in a data set including a wide range of plant species [44].

In the chiltepin population the frequency of potyvirus resistance was moderate, as 21.6% of plants were predicted to be resistant to PVY-LYE84, and 26.0% of *pvr2/eIF4E1* sequences corresponded to resistance alleles (Table 3). Most resistance alleles were identified in SMO populations, and among resistance alleles only *pvr2*^*3*^ and *pvr2*^*4*^ were found in more than one biogeographical province (Fig 2). Interestingly, 55.6% of plants, and 62.5% of *pvr2/eIF4E1* sequences were resistant to PVY-LYE84 in cultivated populations, as compared with 8.4% of plants and 7.8% of sequences in wild ones, and the higher proportion of resistance in cultivated populations held for the three biogeographical provinces in which resistance alleles/plants were found (YUC, SMO and CPA, Table 3). Four out of seven nucleotide sequence haplotypes encoding resistance alleles were found in cultivated populations. Heterozygosity at the *pvr2/eIF4E1* locus was not different in wild or cultivated populations (Table 1, S5 Table), while for SSRs heterozygosity was higher in wild than in cultivated populations, and values were higher than for *pvr2/eIF4E1* [35]. Nucleotide diversity at *pvr2/eIF4E1* was higher in cultivated than in wild populations, whereas a significant decrease in genetic variation at neutral markers in cultivated populations was previously demonstrated in chiltepin [35] as it is commonly observed during plant domestication [26–28]. Also, there was a higher fraction of non-synonymous substitutions in cultivated populations than in wild ones, resulting in *d*_*N*_/*d*_*S*_ ratios indicative of positive selection, as opposed with data from wild populations (Table 2). Last, *D*_*T*_ values were positive for the region between codons 67 and 77, which includes most determinants of potyvirus resistance (Region I in Fig 2), in cultivated but not in wild populations (Fig 1). Thus, all data taken together indicate that selection for potyvirus resistance is stronger in cultivated than in wild chiltepin populations, and results in higher diversification of the *pvr2/eIF4E1* gene. It is noteworthy that both a ~55% frequency of potyvirus resistance and evidence of diversifying selection was found by Charron et al [23] in 25 accessions of *C*. *annuum*, mostly cultivated. High frequency of eIF4E-mediated resistance to the bymoviruses (in family *Potyviridae*) *Barley yellow mosaic virus* and *Barley mild mosaic virus* has also been found in accessions from domesticated barley varieties, with evidence of diversifying selection for resistance [24]. The eIF4E alleles conferring resistance to the potyvirus *Pea seed borne mosaic virus* were only found in domestic pea accessions, in spite of high variability of the locus in wild accessions [25]. So, these reports of other host-virus systems agree with a hypothesis of cultivation-associated selection for resistance at eIF4E.

Although the ecological changes associated with cultivation are considered to favor the incidence of plant pathogens [58,59], which is certainly the case for begomoviruses and other viruses infecting chiltepin in Mexico [38,60], potyvirus incidence in chiltepin did not differ according to habitat (Table 4). However, potyvirus incidence varied less among years in cultivated than in wild populations (Table 4), indicating a more constant challenge of virus infection. Interestingly, in chiltepin populations localized in anthropic environments and tolerated but not cultivated by humans, i.e. “let-standing” populations [35], potyvirus incidence varied temporally as in wild populations (*χ*^2^ = 9.1, *P* = 0.028) strongly suggesting that cultural practices favor a more constant potyvirus prevalence. More significantly, infection in cultivated populations was much more virulent, as 5 times more infected plants showed disease symptoms in cultivated than in wild populations (Table 5), and disease expression can be a good proxy of virulence in plant virus interactions [61–63]. Differences in selection for potyvirus resistance in the wild and under cultivation can be due to human-driven directional selection, as a response to strong symptom expression in cultivated populations, or to natural selection caused by cultivation conditions favoring a more constant and stronger effect of potyvirus infection. The role of natural selection during plant domestication is often overlooked and has been recently emphasized [29]. Also, the shorter generation time in cultivated populations, where chiltepin is managed as an annual crop, as compared with the 4–6 year perennial life span in the wild, could favor a higher selection rate per generation for resistance in the cultivated populations. We cannot at present evaluate the relative role of these contrasting factors on the evolution of potyvirus resistance in chiltepin wild and cultivated populations.

The core structure of the pvr2/eIF4E1 protein would not be affected significantly by the amino acid substitutions found in chiltepin. However, substitutions that uncoupled the pvr2/eIF4E1-VPg interaction, resulting in resistance, were around the cap-binding pocket and strongly affected the electrostatic surface potential at this region, which is reasonable to expect would affect the binding of eIF4E to the cap of cellular mRNAs and, hence its efficiency in translation initiation. Thus, potyvirus resistance would have a cost even if the resistance alleles are fully functional for translation in yeast complementation assays. The location of amino acid substitutions on the protein structure, the low *d*_*N*_/*d*_*S*_ values and the low frequency of resistance alleles in wild chiltepin populations, altogether support a hypothesis of functional constraints translating into costs limiting the evolution of pvr2/eIF4E1 towards potyvirus resistance. *Capsicum* plants carrying an eIF4E1 loss-of-function allele, which could provide evidence on eIF4E1 involvement in development/plant fitness and thus of mutation costs, are not available. A TILLING eIF4E1 knock out allele in cultivated tomato was not associated with obvious developmental defaults under greenhouse conditions [64], although it might be detrimental under more stressful wild conditions. Costs of resistance have been often reported in GFG-like plant-pathogen interactions [10–12,65], but are not a feature of the evolution of pure MA interactions. However, it is considered that real-world host-parasite interactions that mechanistically correspond to a MA model would fall within a continuum between pure MA and GFG models, in which partial infection with less successful parasite multiplication occurs, with correspondingly partial costs of resistance and infectivity [5,7]. This seems indeed to be the case of the pvr2/eIF4E1-mediated interaction between *Capsicum* and potyviruses, as infections largely differ in efficiency and costs of infectivity have been reported [66–68]. Our present results suggest that resistance costs could also determine the evolutionary dynamics of the *Capsicum-Potyvirus* interaction.

The evolution of dominant resistance genes (*R* genes) of plants to cellular pathogens, which are involved in GFG-like interactions, has been analysed extensively. Data indicate that *R* genes are hypermutagenic and often under balancing selection [21,69–72]. The present work focuses on the analysis of the evolution of a recessive resistance gene involved in a MA-like interaction in populations of a wild plant. It also compares evolutionary dynamics between plant populations under different levels of human management. Notably, results show a quite different pattern depending on the level of human management of the habitat. While there is no evidence of high genetic variation or of selection on *pvr2/eIF4E1* in wild chiltepin populations, as often reported for *R* genes [21,69–72], there is evidence of selection on *pvr2/eIF4E1* for potyvirus resistance in the cultivated populations, which is compatible with a hypothesis of balancing selection maintaining *pvr2/eIF4E1* resistance diversity. These major results are perhaps unexpected as cultivation of chiltepin is recent and has not yet resulted in domestication or in obvious phenotypic changes, and the cultivated populations here analysed are not genetically differentiated from sympatric wild ones according to the variation of nuclear SSRs markers [35]. It is widely accepted that human management of plant habitats heavily influence the epidemiology of plant pathogens, including plant viruses [59,73], as has been shown for viruses infecting chiltepin [38,60]. This study shows that human management of the habitat may also have a deep impact on the evolution of plant-pathogen interactions, an underexplored topic in need of more research.

## Materials and Methods

### Chiltepin populations

Chiltepin plants were sampled during the summers of 2007–2010 at different sites over the species distribution in Mexico [35]. Plant samples were collected from chiltepin populations growing in a variety of habitats under different levels of human management [35]. For analyses of the *pvr2/eIF4E1* gene we focused on those from the most extreme levels of human management, i.e. the wild and cultivated populations. Plants grown from seeds in fruits purchased at local markets were also analysed, and were considered here as from wild populations, if (i) the people selling the fruits claimed that they had been collected from local wild chiltepin populations and (ii) after their genetic characterization based on the polymorphisms of nine microsatellite markers [35], those market populations were indeed shown to be related to the local wild populations.

Thus, for analyses of the *pvr2/eIF4E1* gene, we considered a total of 25 populations, 16 wild and 9 cultivated, (S1 Table) from six biogeographical provinces of Mexico: Yucatan (YUC), Eastern side of the Sierra Madre Oriental (SMO), Altiplano Zacatecano-Potosino (AZP), Costa del Pacífico Sur (CPS), Costa del Pacífico (CPA), and Sonora (SON) [74]. A larger set of samples from populations growing in all the habitats (wild, cultivated and let-standing populations) [35] was used to evaluate *Potyvirus* incidence according to biogeographical province, habitat and year of sampling.

### Nucleic acid extraction and amplification of the pvr2/eIF4E1 gene

For analysis of the *pvr2/eIF4E1* gene total nucleic acids were extracted from leaves as in González-Jara et al [35]. The *pvr2/eIF4E1* gene is constituted of 5 exons of 278, 166, 126, 66 and 51 nucleotides (nt), respectively, separated by 4 introns of more than 3500 nt, 110 nt, 1143 nt and 83 nt, respectively [75]. To amplify both introns and exons of the *pvr2/eIF4E1* gene, two different PCRs were run directly on the total nucleic acid extracts, using the Phusion High-Fidelity DNA Polymerase (New England Biolabs, MA, USA). The first PCR was performed with primers F-eIF4E.Full (ATGGCAACAGCTGAAATGGAG) and R-eIF4E.int1 (CCCCGAGAATCTTAGTAGCTCA), designed to amplify a 756 nt fragment including *pvr2/eIF4E1* exon 1 and the 5’ most 403 nt of intron 1. Conditions for this PCR were 98°C for 30 sec, and 35 cycles of 98°C for 10 sec, 56°C for 30 sec and 72°C for 25 sec. The second PCR was performed using primers F-eIF4E.ex2 (TGCTTACAATAATATCCACCACCC) and R-eIF4E.3’UTR (CACAAGGTACTCAAACCAGAAGC), designed to amplify a 1848 nt fragment including the four other exons of *pvr2/eIF4E1* and introns 2 to 4. Conditions for this PCR were 98°C for 30 sec, and 35 cycles of 98°C for 10 sec, 54°C for 30 sec and 72°C for 1 min. Primers F-eIF4E.Full and R-eIF4E.int1 were also used to obtain the full nucleotide sequence of the amplicon from the first PCR. To determine the nucleotide sequence of the amplicon from the second PCR, primers F-eIF4E.ex2, R-eIF4E.int3 (CCCCTTCATCTATAAGCATATTTC), F-eIF4E.int3end (GATGGTCTCAAGGGTTATGTGTC) and R-eIF4E.3’UTR were used, in order to obtain the complete sequence of exons 2, 3, 4 and 5, and of introns 2 and 4, and two partial sequences of intron 3 (5’ fragment: 293 nt; 3’ fragment: 547 nt). The *pvr2/eIF4E1* coding sequence was then deduced from the exon sequences.

Sequence analyses identified plants heterozygous for the *pvr2/eIF4E1* gene. Sequence determination in heterozygotes was done after RT-PCR amplification of *pvr2/eIF4E1* coding sequences and/or cloning of the DNA amplicons in pCRII (TA Cloning Kit Dual Promoter, *Invitrogen*, Carlsbad, CA, USA). RT-PCR amplification of *pvr2/eIF4E1* coding sequences was also used to identify the *pvr2/eIF4E1* allele(s) present in virus-inoculated plants (see below). In this case, the RT step was performed with the SuperScript III Reverse Transcriptase (*Invitrogen*) according to the manufacturer’s recommendations using primer R-eIF4E.3’UTR, followed by a PCR amplifying the cDNA corresponding to the full coding sequence of *pvr2/eIF4E1* with the primers F-eIF4E.Full and R-eIF4E.3’UTR (PCR conditions: 98°C for 30 sec, and 35 cycles of 98°C for 10 sec, 53°C for 30 sec and 72°C for 25 sec).

### Population genetic analyses

Nucleotide sequences were aligned to maintain the reading frame using CLUSTAL-W [76] as implemented in Mega 6 [77]. Differences in heterozygous plants at the *pvr2*/*eIF4E1* locus, in haplotype richness and in resistance frequency between populations, regions or habitat were assessed by the analysis of contingency tables using the Fisher exact test. Genetic diversity within and between populations, biogeographical provinces or levels of human management were estimated using the Kimura 2-parameter model, with standard errors of each measure based on 1000 replicate bootstraps, as implemented in Mega 6. Differences in nucleotide diversity of the virus populations among biogeographical provinces and between habitats were tested by analysis of molecular variance (AMOVA), as implemented in Arlequin v. 5.3.1.2 [78]. Differences in *d*_N_/*d*_S_ values were considered to be significant if the mean value of one estimate fell outside of the 95% CI values of another, indicating that these *d*_N_/*d*_S_ values were drawn from different distributions. AMOVA calculates the *F*_*ST*_ index explaining the between-groups fraction of total genetic diversity. Significance of these differences was obtained by performing 1000 permutations. Tajima’s *D* (*D*_*T*_) and sliding window analyses were conducted using DnaSP v. 5.10 [79].

Mantel correlation tests between geographic and genetic distance matrices were performed to test the isolation-by-distance hypothesis [80] in wild chiltepin populations using the web service http://ibdws.sdsu.edu/~ibdws/ [81]. We used the geographic distance matrices obtained in González-Jara et al [35]. Geographical and genetic distances between pairs of populations were log transformed, and 1000 permutations were performed to assess the significance of the correlations.

### Nucleotide sequence analyses

We used the median-joining network method implemented in the Network version 4.611 software (available at www.fluxus-engineering.com) [82] to reconstruct the minimum spanning network (MSN) connecting all chiltepin *pvr2/eIF4E1* alleles identified at the amino acid level. Phylogenetic relationships were reconstructed by the Neighbor-Joining method as implemented in Mega 6 [77] and incorporating the best-fitted nucleotide substitution model (F81 model) determined by jModelTest 0.1.1 [83]. The sequence of the *Potyvirus* susceptibility allele *pot-1*^+^ from tomato (*Solanum lycopersicum*, accession number AY723733) was used as outgroup. Phylogenies were also reconstructed by Maximum Likelihood and by Maximum Parsimony using Subtrees Pruning and Regrafting method as implemented in Mega 6 with similar results.

The ratio of non-synonymous (*d*_*N*_) to synonymous (*d*_*S*_) substitutions over the *pvr2/eIF4E1* coding sequences from chiltepin populations was estimated by the Pamilo-Bianchi-Li method as implemented in Mega 6. The *d*_*N*_/*d*_*S*_ ratio was also estimated at individual codons in the *pvr2/eIF4E1* coding sequences, using different methods implemented in the HYPHY program (SLAC, Single Likelihood Ancestor Counting; FEL, Fixed Effects Likelihood; IFEL, Internal Fixed Effects Likelihood; REL, Random Effects Likelihood; FUBAR, Fast Unbiased Bayesian Approximation) [84–87] to determine whether each of the 228 codons of *pvr2/eIF4E1* were under negative (*d*_*N*_/*d*_*S*_<1), neutral (*d*_*N*_/*d*_*S*_ = 1), or positive (*d*_*N*_/*d*_*S*_>1) selection. These analyses were performed after confirmation of the absence of recombinant sequences in our dataset by two methods implemented in the HYPHY program (SBP, Single Breakpoint Recombination; GARD, Genetic Algorithms for Recombination Detection) [86] and using the tree topology previously obtained for *pvr2/eIF4E1*.

### Functional characterization of pvr2/eIF4E1 alleles in yeast

The *Saccharomyces cerevisiae* strain JO55 [cdc33-D LEU2 leu2 ura3 his3 trp1 ade2 (YCp33supex-h4E URA3)] [88], carrying a disrupted endogenous eIF4E gene (cdc33), was used as in Charron et al [23] to verify the functionality of the *pvr2/eIF4E1* allelic variants identified in chiltepin populations. The coding sequence of the *pvr2*^+^ allele was cloned into the p424GBP/TRP1 glucose-dependent vector, and all *pvr2/eIF4E1* allelic variants were obtained by mutagenesis of this construct using the QuikChange Site-Directed Mutagenesis Kit (*Stratagene*, Agilent Technologies, Santa Clara, CA, USA). Each construct was sequenced to confirm the presence of the introduced mutations and then independently used to transform *S*. *cerevisiae* strain JO55. After transformation, yeast cells were grown in appropriate selective nutrient drop-out media containing 2% glucose. Control transformations were performed with no DNA (untransformed yeast JO55) and empty p424GBP/TRP1 plasmids (negative controls), and with p424GBP/TRP1::At-eIF4E (eIF4E form of *Arabidopsis thaliana*, At4g18040) as a positive control. After transformation, yeast colonies were grown to stationary phase, were suspended in sterile water, and then were adjusted to an OD_600nm_ of 5.10^−2^, 5.10^−3^, and 5.10^−4^ before spotting 10 μl aliquots onto the appropriate media in order to test for their ability to complement the lack of endogenous eIF4E at 30°C [89]. For each *pvr2/eIF4E1* allelic variant, 3 independent colonies were randomly selected to perform the complementation assay.

The Matchmaker GAL4 two-hybrid system 3 (*Clontech*, Mountain View, CA, USA) was used according to the manufacturer’s recommendations to evaluate the interaction of the proteins encoded by the *pvr2/eIF4E1* allelic variants with the potyviral VPg. The constructs previously developed by Charron et al [23] were used. The *eIF4E1*/*pvr2*^+^ coding sequence was cloned in-frame with the GAL4 activation domain into the pGADT7 vector (*Clontech*, Mountain View, CA, USA), and all *pvr2/eIF4E1* allelic variants were obtained by mutagenesis with the QuikChange Site-Directed Mutagenesis Kit (*Stratagene*). All the constructs were sequenced to confirm the presence of the introduced mutations before yeast transformation. The VPg of PVY (avirulent isolate LYE84) [90] and of TEV (avirulent isolate HAT) [48] were cloned in-frame with the GAL4 binding domain into the pGBKT7 vector, respectively [23]. The pGADT7- and pGBKT7-derived vectors were transformed into AH109 and Y187 yeast strains, respectively, which contain two independent reporter genes, HIS3 and ADE2, to confer histidine and adenine auxotrophy, respectively, driven by hybrid GAL4 promoters. After yeast mating, double-transformed yeast colonies were grown to stationary phase, were suspended in sterile water, and then were adjusted to an OD_600nm_ of 5.10^−2^ before spotting 10 μl aliquots onto various selective media including synthetic medium lacking leucine and tryptophan (hereafter named -LW) and medium lacking leucine, tryptophan and histidine (-LWH). Plates were incubated at 30°C, and yeast growth was checked daily from 2 to 7 days after spotting. The yeast growth on the selective–LWH medium reflects the pvr2/eIF4E1-VPg physical interactions. Empty pGADT7 and pGBKT7 vectors were used as negative controls and interaction between murine p53 and SV40 large T antigen as positive controls. Three independent yeast-two hybrid assays were performed, in which 3 independent colonies of each pvr2/eIF4E1-VPg combination were randomly selected.

For complementation and yeast-two hybrid assays, growth intensities were monitored with ImageJ software [91], and raw data were normalized to positive and negative controls and expressed as a percentage of the growth of the reference yeast colonies (transformed with p424GBP/TRP1::*eIF4E1*/*pvr2*^+^ for complementation assays, and co-transformed with pGADT7::*eIF4E1*/*pvr2*^+^ and pGBKT7::VPg-PVY for yeast two-hybrid assays) as previously described in Hébrard et al [92].

### Modelling of pvr2/eIF4E1 structure and Poisson-Boltzmann electrostatic potentials

The secondary structure of eIF4E proteins used in this study from *Capsicum annuum pvr2*^+^ allele; *Triticum aestivum*, 2IDR; *Pisum sativum*, 2WMC; and the mammalian eIF4Es used as outgroup from *Homo sapiens*, PDB ID: 4DT6; *Mus musculus*, 1L8B [54,93–95] was predicted using the server NPS, which deduced the consensus secondary structure of protein from 12 different methods (http://npsa-pbil.ibcp.fr) [96].

The tertiary structure of all the pvr2/eIF4E1 alleles identified in chiltepin populations was modelled with the Iterative Threading ASSEmbly Refinement (I-TASSER) hybrid method [97–99]. Starting from an amino acid sequence, I-TASSER first generates 3D atomic models from multiple threading alignments and iterative structure assembly conducted by Monte Carlo simulations under an optimized knowledge-based force field. The lowest free-energy conformations are identified by structure clustering and final atomic structure models are constructed from the low-energy conformations by means of a two-step atomic-level energy minimization approach. The correctness of the models is assessed by a confidence score (C-score) and a measure of structural similarity (TM-score). In all cases, the 3D structures were constructed from scratch without resorting to previous models of other alleles. Among the five models predicted by I-TASSER, that having the best values of both C-score and TM-score was finally selected. The main pvr2^+^ structure had C-score = 0.09 (C-score is typically in the [−5, 2] range, with a higher value meaning a model with higher confidence) and TM-score = 0.73 ± 0.11 (a TM-score > 0.5 indicates a model of correct topology). For the remaining alleles, C-score ranged from -1.57 and +0.28 and TM-score ranged between 0.52 ± 0.15 and 0.75 ± 0.10 so that all the 3D models presented here for the different pvr2/eIF4E1 alleles may be considered as having significant confidence and being topologically correct.

The 3D model structures were first visualized and analyzed with Swiss-PdbViewer 4.1.0 [100], software which was also used for rendering van der Waals (VdW) surfaces, obtaining pairwise structural superpositions and computing the corresponding root mean square deviation (RMSD) values. All structure models of pvr2/eIF4E1 alleles showed an N-terminal unstructured segment spanning the first 45–50 residues in their amino acid sequences. To further assess this result, we applied the following predictors of protein disorder: DisEMBL [101], DISOPRED [102], and IUPred [103] to the amino acid sequence of the main pvr2^+^ allele. Given that they employ disparate algorithms based on rather different assumptions, their close agreement in predicting disorder for segments 1–44 (DisEMBL), 1–50 (DISOPRED), and 1–45 (IUPred) lend further support to the structural models generated by I-TASSER.

Poisson-Boltzmann (PB) electrostatic potentials mapped onto the protein surface of all the pvr2/eIF4E1 alleles were computed by solving the PB equation with APBS 1.4 [104] using AMBER99 [105] atomic charges and radii assigned with PDB2PQR 1.7 [106]. The nonlinear PB equation was solved at 298.15 K and 0.150 M ionic concentration in sequential focusing multigrid calculations in 3D meshes of 160^3^ or 192^3^ points with step sizes about 0.35 or 0.50 Å depending on the particular pvr2/eIF4E1 allele. Dielectric constants 4 for proteins and 78.54 for water were used. The output of PB electrostatic potentials thus computed were obtained in scalar OpenDX format and these numerical meshes were then mapped onto molecular surfaces of proteins and rendered with PyMOL 1.6 (PyMOL, Schrodinger, LLC). PB electrostatic potential values are given in units of *kT* per unit charge (*k*, Boltzmann's constant and *T*, absolute temperature).

### Potyvirus resistance evaluation and potyvirus detection in Chiltepin populations

All plants were grown under greenhouse conditions and transferred into growth chambers before inoculation (16h light/8h dark; 24°C/18°C). Chiltepin plants were mechanically inoculated at the cotyledon stage with PVY-LYE84 (pathotype PVY-0) and TEV-HAT [48,90] as previously described [107]. The *C*. *annuum* accessions Yolo Wonder (*pvr2*^+^ homozygous, susceptible to PVY-LYE84 and TEV-HAT) and Florida (*pvr2*^2^ homozygote, resistant to PVY-LYE84 and TEV-HAT) were used as susceptible and resistant controls, respectively. Plants mock-inoculated with buffer were used as negative controls. Systemic infection was assessed by determining the presence/absence of symptoms on non-inoculated leaves and confirmed by DAS-ELISA using PVY or TEV antibodies.

Infection by *Potyvirus* species in natural chiltepin populations was detected by DAS-ELISA, using the complete kit of detection PSA 27200/0288 according to the manufacturer’s recommendation (*AGDIA*, Elkhart, IN, USA). This kit is based on the broad reactivity of a monoclonal antibody reacting to a highly conserved amino acid sequence on the coat protein of the *Potyvirus* genus. A total of 955 plants from 24 wild and cultivated populations were analysed in this way, plus 238 plants from let-standing populations. Differences in potyvirus incidence or symptom frequency in infected plants were assessed by the analysis of contingency tables using the Fisher exact test. The presence of virus in the ELISA-positive samples was confirmed by RT-PCR using the potyvirus-specific degenerated primers designed by Zheng et al [108], which amplify a region of the NIb gene (positions 7619–7968) highly conserved between *Potyvirus* species. Once the *Potyvirus* species was identified by NIb sequencing, species-specific primers bordering the VPg and the CP were designed. These primers were: for PepMoV, F-PepMoV.VPg: GTGCATCACCAGTCCAAGTCTT and R-PepMoV.VPg: CAGTCAACTTGCAAACAGTTTGG, F-PepMoV.CP: GCTGACTTGGCATCTGAAGGA and R-PepMoV.CP: TTCATCCCAGAGACCACATCAG; for TEV-like virus, F-TEVlike.VPg: GTATCATCCAAGACTTCAATCACCTGGAAAC and R-TEVlike.VPg: GATGTTGTGTGCCCATCAGATTCATTC, F-TEVlike.CP: CACAGCTTGCAGARGAAGGAAAGGC and R-TEVlike.CP: CTTAAAAGCGGAAAGCAAAGACACGC).

## Supporting Information

S1 Table Chiltepin populations analysed and number of *pvr2/eIF4E1* sequences obtained in this study. (DOCX)

S2 Table Frequency of heterozygous plants for the *pvr2/eIF4E1* coding sequences in chiltepin populations according to geographical provinces and habitats. (DOCX)

S3 Table Genetic diversity of *pvr2/eIF4E1* exons and introns in chiltepin populations according to geographical provinces and habitats. (DOCX)

S4 Table Genetic differentiation of the *pvr2/eIF4E1* coding sequences between biogeographical provinces. (DOCX)

S5 Table Zygosity at the *pvr2/eIF4E1* locus and identification of susceptible and resistant plants in chiltepin populations. (DOCX)

S1 Fig Geographic and genetic distance in wild chiltepin populations at the *pvr2/eIF4E1* locus. Log-transformed data are presented.
(TIF)

S2 Fig Complementation of yeast strain JO55 with *pvr2/eIF4E1* coding sequences identified in chiltepin populations. Control -: negative control corresponding to yeast colonies transformed with empty p424GBP/TRP1 plasmids.
(TIF)

S3 Fig Effects of the mutations identified in pvr2/eIF4E1 protein on the pvr2/eIF4E1-VPg:PVY interactions evaluated. Yeast growth was evaluated by yeast two hybrid assays and was expressed by percentage of the yeast growth on the selective medium (-LWH) compared to the reference yeast colonies co-transformed with pGADT7::*pvr2*^+^ and pGBKT7::VPg-PVY; standard errors were obtained after 3 replications of the yeast two-hybrid assays in which 3 independent colonies of each pvr2/eIF4E1-VPg:PVY combination were randomly selected.
(TIF)

S4 Fig Sequence alignment of pvr2/eIF4E1 alleles identified in chiltepin populations and localisation of the polymorphic positions (orange), sites involved in the *Potyvirus* resistance (purple) and in the m7GTP binding cap recognition (green) and domain of eIF4G interaction (blue). (TIF)

S5 Fig Phylogenetic relationships between *Capsicum annuum pvr2*^+^ allele and 4 crystallized eIF4E (A), comparison of the secondary structure of these proteins (B) and disorder prediction in the pvr2/eIF4E1 protein (C). *: localisation of the polymorphic positions identified in the pvr2/eIF4E1 protein of chiltepins.
(TIF)

S6 Fig Superposition of the 3D-model generated independently of the pvr2/eIF4E1 alleles identified in chiltepin populations. (A) superposition of the models; (B) *pvr2*^+^ (green) and *pvr1*^+^ (orange), superposition: RMSD_backbone_ = 1.050 Å; (C) *pvr2*^+^ (green) and *pvr2*^1^ (orange), superposition: RMSD_backbone_ = 1.107 Å; (D) *pvr2*^+^ (green) and *pvr2*^2^ (orange), superposition: RMSD_backbone_ = 0.978 Å; (E) *pvr2*^+^ (green) and *pvr2*^4^ (orange), superposition: RMSD_backbone_ = 1.090 Å; (F) *pvr2*^+^ (green) and *pvr2*^7^ (orange), superposition: RMSD_backbone_ = 0.999 Å; (G) *pvr2*^+^ (green) and *pvr2*^9^ (orange), superposition: RMSD_backbone_ = 0.999 Å; (H) *pvr2*^+^ (green) and *pvr2*^17^ (orange), superposition: RMSD_backbone_ = 0.602 Å; (I) *pvr2*^+^ (green) and *pvr2*^23^ (orange), superposition: RMSD_backbone_ = 0.633 Å; (J) *pvr2*^+^ (green) and *pvr2*^24^ (orange), superposition: RMSD_backbone_ = 0.692 Å; (K) *pvr2*^+^ (green) and *pvr2*^25^ (orange), superposition: RMSD_backbone_ = 0.716 Å.
(TIF)

S7 Fig Phylogenetic relationships between *Potyvirus* species and location of the potyviruses infecting chiltepin populations. The presented phylogeny was reconstructed based on partial NIb (304nt) by the NJ method, bootstrap values (1000 replicates) are indicated on the nodes. Grey boxes mentioned the potyvirus sequences identified in chiltepin populations.
(TIF)

S8 Fig Differences in the VPg protein sequence between TEV and TEV-like potyviruses. TEV (Consensus): Consensus sequence obtained with 20 world-wide TEV isolates (accession numbers: L38714, M11458, M15239.1, NC_001555.1, DQ986288.1, EF470242.2, JN711120.1, EU334794.1, EU334793.1, EU334792.1, EU334791.1, EU334790.1, 334789.1, EU334788.1, EU334787.1, EU334786.1, EU334785.1, EU334784.1, EU334783.1, JX512812.1); TEV-Mex21 (RB *pvr1*): Sequence of a *pvr1* resistance-breaking TEV isolate (KM282188); TEV-N (RB *pvr1*^2^): Sequence of a *pvr1*^2^ resistance-breaking TEV isolate (KM282189); Grey boxes: Mutations putatively involved in the resistance-breaking process; Red letters: discriminating position between TEV-like and TEV.
(TIF)

S9 Fig Phylogeny of *pvr2/eIF4E1* coding sequences in the genus *Capsicum*. The presented phylogeny was reconstructed by the NJ method, bootstrap values (1000 replicates) are indicated on the nodes. hapA to hapQ are *pvr2/eIF4E1* haplotypes identified in chiltepin populations; *pvr2*^*+*^ and *pvr2*^1^ to *pvr2*^9^ are *pvr2/eIF4E1* haplotypes described in [23]; *pvr1*^+^, *pvr1* and *pvr1*^2^ are described in [22]; *pvr2*^10^ to *pvr2*^22^ are described in [45]; *pot1*^*+*^: *Potyvirus* susceptibility allele from tomato (accession number AY723733). Green label: susceptible allele; red label: resistant allele; grey label: not characterized allele.
(TIF)

S10 Fig Phylogeny of *pvr2/eIF4E1* coding sequences in the genus *Capsicum* based on the first and the second positions of the codons. The presented phylogeny was reconstructed by the NJ method, bootstrap values (1000 replicates) are indicated on the nodes. hapA to hapQ are *pvr2/eIF4E1* haplotypes identified in chiltepin populations; *pvr2*^*+*^ and *pvr2*^1^ to *pvr2*^9^ are *pvr2/eIF4E1* haplotypes described in [23]; *pvr1*^+^, *pvr1* and *pvr1*^2^ are described in [22]; *pvr2*^10^ to *pvr2*^22^ are described in [45]; *pot1*^*+*^: *Potyvirus* susceptibility allele from tomato (accession number AY723733). Green label: susceptible allele; red label: resistant allele; grey label: not characterized allele.
(TIF)

S11 Fig Phylogeny of *pvr2/eIF4E1* coding sequences in the genus *Capsicum* based only on the third positions of the codons. The presented phylogeny was reconstructed by the NJ method, bootstrap values (1000 replicates) are indicated on the nodes. hapA to hapQ are *pvr2/eIF4E1* haplotypes identified in chiltepin populations; *pvr2*^*+*^ and *pvr2*^1^ to *pvr2*^9^ are *pvr2/eIF4E1* haplotypes described in [23]; *pvr1*^+^, *pvr1* and *pvr1*^2^ are described in [22]; *pvr2*^10^ to *pvr2*^22^ are described in [45]; *pot1*^*+*^: *Potyvirus* susceptibility allele from tomato (accession number AY723733). Green label: susceptible allele; red label: resistant allele; grey label: not characterized allele.
(TIF)
