# Supplementary material for: Human Management of a Wild Plant Modulates the Evolutionary Dynamics of a Gene Determining Recessive Resistance to Virus Infection
Source: PLoS Genet. 2016 Aug 4;12(8):e1006214. doi: 10.1371/journal.pgen.1006214 (PMC4973933; doi:10.1371/journal.pgen.1006214)
Supplement: S1 Table — (DOCX) [file pgen.1006214.s001.docx]

| **S1 Table.** Chiltepin populations analysed and number of *pvr2/eIF4E1* sequences obtained from each of them in this study ^a)^. | | | | | | | | |
| --- | --- | --- | --- | --- | --- | --- | --- | --- |
| **Code** | **Location** | **Region** | **Habitat** | **Latitude** | **Longitude** | **Year** | **N_plants_** | **N_Seq_** |
| BAT-W | Batopilas | Sonora (SON) | W | 27.027 | -107,739 | 2009 | 2 | 2 (2LM) |
| HER-C | Hermosillo | Sonora (SON) | C | 29.013 | -111,134 | 2009 | 2 | 2 |
| MAU-W | Los Mautos | Sonora (SON) | W | 28.635 | -110,188 | 2009 | 4 | 4 |
| MOC-W | Moctezuma | Sonora (SON) | W | 29.571 | -110,002 | 2009 | 6 (2LM) | 6 (2LM) |
| SJA-W | San Javier | Sonora (SON) | W | 28.600 | -109,716 | 2007 | 4 | 4 |
| SJP-W | San Juan de Pimes | Sonora (SON) | W | 27.116 | -110,131 | 2009 | 2 (2LM) | 2 (2LM) |
| TEM-C | Temporal | Sonora (SON) | C | 28.715 | -110,351 | 2009 | 2 | 2 |
| ELO-W | Elota | Costa del Pacifico (CPA) | W | 24.014 | -106,706 | 2009 | 6 (2LM) | 6 (2LM) |
| HUJ-W | El Huajote | Costa del Pacifico (CPA) | W | 23.106 | -106,116 | 2009 | 4 | 4 |
| HUJ-C | El Huajote | Costa del Pacifico (CPA) | C | 23.127 | -106,057 | 2009 | 2 | 2 |
| LIB-C | La Libertad | Costa del Pacifico (CPA) | C | 21.593 | -105,173 | 2009 | 2 | 2 |
| PEL-W | Puente Elota | Costa del Pacifico (CPA) | W | 23.954 | -106,726 | 2009 | 4 | 4 |
| PLC-W | Playa Ceuta | Costa del Pacifico (CPA) | W | 23.919 | -106,887 | 2009 | 2 (2LM) | 3 (3LM) |
| BER-W | Bernal | Altiplano Zacatecano-Potosino (AZP) | W | 20.910 | -99,826 | 2009 | 6 (2LM) | 8 (2LM) |
| CER-W | Cerritos | Altiplano Zacatecano-Potosino (AZP) | W | 22.451 | -100,239 | 2009 | 6 (2LM) | 9 (4LM) |
| CER-C | Cerritos | Altiplano Zacatecano-Potosino (AZP) | C | 22.448 | -100,244 | 2009 | 2 | 2 |
| TUL-W | Tula | Altiplano Zacatecano-Potosino (AZP) | W | 23.001 | -99,659 | 2009 | 4 | 5 |
| PVE-C | Puerto Verde | Sierra Madre Oriental (SMO) | C | 21.912 | -99,423 | 2009 | 5 | 6 |
| TLA-W | Tlacuapa | Sierra Madre Oriental (SMO) | W | 21.418 | -98,945 | 2007 | 4 | 4 |
| TLA-C | Tlacuapa | Sierra Madre Oriental (SMO) | C | 21.417 | -98,947 | 2009 | 8 | 11 |
| XIL-W | Xilitla | Sierra Madre Oriental (SMO) | W | 21.384 | -98,992 | 2009 | 2 (2LM) | 2 (2LM) |
| HUA-W | Huatulco | Costa del Pacifico Sur (CPS) | W | 15.795 | -96,053 | 2009 | 4 | 4 |
| HUA-C | Huatulco | Costa del Pacifico Sur (CPS) | C | 15.800 | -96,055 | 2009 | 2 | 2 |
| DZI-W | Dzibilchaltun | Yucatan (YUC) | W | 21.092 | -89,595 | 2009 | 10 | 10 |
| CHO-C | Cholul | Yucatan (YUC) | C | 21.053 | -89,558 | 2009 | 2 | 3 |
| **Total** |  |  |  |  |  |  | **97** (14LM) | 1. (19LM) |

^a)^ W and C: wild and cultivated populations, respectively; N_Plants_ and N_Seq_: number of chiltepin plants and *pvr2/eIF4E1* sequences analysed, respectively; LM: number of plants or pvr2/eIF4E1 sequences obtained from the local market chiltepin populations considered in this study as wild populations.
