# Supplementary material for: Human Management of a Wild Plant Modulates the Evolutionary Dynamics of a Gene Determining Recessive Resistance to Virus Infection
Source: PLoS Genet. 2016 Aug 4;12(8):e1006214. doi: 10.1371/journal.pgen.1006214 (PMC4973933; doi:10.1371/journal.pgen.1006214)
Supplement: S2 Table — (DOCX) [file pgen.1006214.s002.docx]

**S2 Table.** Frequency of plants heterozygous at *pvr2/eIF4E1* in chiltepin populations according to geographical provinces and habitats ^a)^.

|  |  |  |  |  |
| --- | --- | --- | --- | --- |
|  | **N total** | **N homo.** | **N hetero.** | **% hetero.** |
| **SON** | 22 | 22 | 0 | 0.0 |
| **CPA** | 20 | 19 | 1 | 5.0 |
| **AZP** | 18 | 12 | 6 | 33.3 |
| **SMO** | 19 | 15 | 4 | 21.1 |
| **CPS** | 6 | 6 | 0 | 0.0 |
| **YUC** | 12 | 11 | 1 | 8.3 |
| **W** | 70 | 63 | 7 | 10.0 |
| **C** | 27 | 22 | 5 | 18.5 |
| **Total** | **97** | **85** | **12** | **12.4** |

^a)^ N total: total number of plants analysed; N homo. and N hetero.: number of homozygous and heterozygous plants for *pvr2/eIF4E1*, respectively.
