# Supplementary material for: Human Management of a Wild Plant Modulates the Evolutionary Dynamics of a Gene Determining Recessive Resistance to Virus Infection
Source: PLoS Genet. 2016 Aug 4;12(8):e1006214. doi: 10.1371/journal.pgen.1006214 (PMC4973933; doi:10.1371/journal.pgen.1006214)
Supplement: S3 Table — (DOCX) [file pgen.1006214.s003.docx]

**S3 Table.** Genetic diversity of *pvr2/eIF4E1* exons and introns in chiltepin populations according to geographical provinces and habitats ^a)^.

|  |  |  |  |  |  |  |  |
| --- | --- | --- | --- | --- | --- | --- | --- |
| **Groups** | **π** (pvr2 exons) | **π** (pvr2 introns) | **π** (full pvr2 gene) | **π** (pvr2 intron1) | **π** (pvr2 intron2) | **π** (pvr2 intron3) | **π** (pvr2 intron4) |
| **SON** | 0.00098 ± 0.00070 | 0.00454 ± 0.00135 | 0.00339 ± 0.00098 | 0.00782 ± 0.00287 | 0.00228 ± 0.00237 | 0.00257 ± 0.00127 | 0.01239 ± 0.00893 |
| **CPA** | 0.00125 ± 0.00066 | 0.00714 ± 0.00128 | 0.00543 ± 0.00108 | 0.01032 ± 0.00320 | 0.00000 ± 0.00000 | 0.00571 ± 0.00173 | 0.00000 ± 0.00000 |
| **AZP** | 0.00110 ± 0.00080 | 0.00099 ± 0.00042 | 0.00089 ± 0.00039 | 0.00221 ± 0.00136 | 0.00135 ± 0.00146 | 0.00074 ± 0.00063 | 0.00000 ± 0.00000 |
| **SMO** | 0.00333 ± 0.00127 | 0.00020 ± 0.00014 | 0.00100 ± 0.00027 | 0.00160 ± 0.00094 | 0.00000 ± 0.00000 | 0.00000 ± 0.00000 | 0.00000 ± 0.00000 |
| **CPS** | 0.00079 ± 0.00077 | 0.00519 ± 0.00147 | 0.00381 ± 0.00108 | 0.00310 ± 0.00171 | 0.00000 ± 0.00000 | 0.00585 ± 0.00190 | 0.00000 ± 0.00000 |
| **YUC** | 0.00390 ± 0.00160 | 0.00140 ± 0.00077 | 0.00158 ± 0.00074 | 0.00831 ± 0.00309 | 0.00000 ± 0.00000 | 0.00320 ± 0.00140 | 0.00000 ± 0.00000 |
| **W** | 0.00359 ± 0.00115 | 0.00606 ± 0.00112 | 0.00512 ± 0.00109 | 0.00915 ± 0.00263 | 0.00085 ± 0.00060 | 0.00491 ± 0.00136 | 0.00632 ± 0.00473 |
| **C** | 0.00347 ± 0.00149 | 0.00598 ± 0.00093 | 0.00521 ± 0.00105 | 0.00710 ± 0.00214 | 0.00080 ± 0.00081 | 0.00550 ± 0.00138 | 0.00000 ± 0.00000 |
| **overall** | 0.00360 ± 0.00132 | 0.00655 ± 0.00130 | 0.00561 ± 0.00106 | 0.00933 ± 0.00262 | 0.00082 ± 0.00062 | 0.00586 ± 0.00157 | 0.00415 ± 0.00302 |
|  |  |  |  |  |  |  |  |

^a)^ Genetic diversity (π) of *pvr2/eIF4E1* coding sequence (pvr2 exons), *pvr2/eIF4E1* concatenated introns (pvr2 introns), full-length *pvr2/eIF4E1* gene (full pvr2 gene), and individual *pvr2/eIF4E1* introns (pvr2 intron1 to pvr2 intron4).
