# Supplementary material for: Human Management of a Wild Plant Modulates the Evolutionary Dynamics of a Gene Determining Recessive Resistance to Virus Infection
Source: PLoS Genet. 2016 Aug 4;12(8):e1006214. doi: 10.1371/journal.pgen.1006214 (PMC4973933; doi:10.1371/journal.pgen.1006214)
Supplement: S4 Table — (DOCX) [file pgen.1006214.s004.docx]

**S4 Table.** Genetic differentiation of the *pvr2/eIF4E1* coding sequences between biogeographical provinces ^a)^.

| **A. All populations** | | | | | | | |
| --- | --- | --- | --- | --- | --- | --- | --- |
|  |  |  |  | *P values* |  |  |  |
|  |  | **SON** | **CPA** | **AZP** | **SMO** | **CPS** | **YUC** |
|  | **SON** |  | 0.00000 | 0.00000 | 0.00000 | 0.02703 | 0.00000 |
|  | **CPA** | 0.16795 |  | 0.00000 | 0.00000 | 0.09910 | 0.00000 |
| *Fst* | **AZP** | 0.74141 | 0.66521 |  | 0.00000 | 0.00000 | 0.00000 |
|  | **SMO** | 0.58314 | 0.48861 | 0.57893 |  | 0.00000 | 0.00000 |
|  | **CPS** | 0.21896 | 0.11289 | 0.74327 | 0.50750 |  | 0.00901 |
|  | **YUC** | 0.38308 | 0.26525 | 0.55105 | 0.29913 | 0.26297 |  |
|  |  |  |  |  |  |  |  |
| **B. Wild populations** | | | | | | | |
|  |  |  |  | *P values* |  |  |  |
|  |  | **SON** | **CPA** | **AZP** | **SMO** | **CPS** | **YUC** |
|  | **SON** |  | 0.00901 | 0.00000 | 0.00000 | 0.49550 | 0.00000 |
|  | **CPA** | 0.14981 |  | 0.00000 | 0.00000 | 0.99099 | 0.00000 |
| *Fst* | **AZP** | 0.72436 | 0.74613 |  | 0.00000 | 0.00000 | 0.00000 |
|  | **SMO** | 0.58315 | 0.60150 | 0.58355 |  | 0.00901 | 0.01802 |
|  | **CPS** | 0.04425 | 0.10334 | 0.73478 | 0.41983 |  | 0.05405 |
|  | **YUC** | 0.44965 | 0.46234 | 0.64658 | 0.40910 | 0.32562 |  |

| **C. Cultivated populations** | | | | | | | |
| --- | --- | --- | --- | --- | --- | --- | --- |
|  |  |  |  | *P values* |  |  |  |
|  |  | **SON** | **CPA** | **AZP** | **SMO** | **CPS** | **YUC** |
|  | **SON** |  | 0.08264 | 0.02479 | 0.00000 | 0.07438 | 0.01653 |
|  | **CPA** | 0.37032 |  | 0.07438 | 0.00000 | 0.01653 | 0.09917 |
| *Fst* | **AZP** | 0.86013 | 0.52991 |  | 0.02479 | 0.42149 | 0.09091 |
|  | **SMO** | 0.58467 | 0.35356 | 0.62416 |  | 0.04959 | 0.57025 |
|  | **CPS** | 0.68023 | 0.44987 | 1.00000 | 0.62416 |  | 0.15702 |
|  | **YUC** | 0.75500 | 0.41403 | 0.79888 | 0.04907 | 0.79888 |  |

^a)^ Values of *F_ST_* (below the diagonal) and significance (*P*, above the diagonal) of the *pvr2/eIF4E1* coding sequences in overall chiltepin populations (A), in wild chiltepin populations (B) and in cultivated populations (C). Region indicates the biogegraphical province.
