# Supplementary material for: Human Management of a Wild Plant Modulates the Evolutionary Dynamics of a Gene Determining Recessive Resistance to Virus Infection
Source: PLoS Genet. 2016 Aug 4;12(8):e1006214. doi: 10.1371/journal.pgen.1006214 (PMC4973933; doi:10.1371/journal.pgen.1006214)
Supplement: S5 Table — (DOCX) [file pgen.1006214.s005.docx]

**S5 Table.** Zygosity at the *pvr2/eIF4E1* locus and identification of susceptible and resistant plants in chiltepin populations ^a)^.

|  | **Population** | **N plant** | **N hetero.** | **N s/s** | **N R/s** | **N R/R** |
| --- | --- | --- | --- | --- | --- | --- |
| **SON** | BAT-W | 2 | 0 | 2 | - | - |
| **SON** | HER-C | 2 | 0 | 2 | - | - |
| **SON** | MAU-W | 4 | 0 | 4 | - | - |
| **SON** | MOC-W | 6 | 0 | 6 | - | - |
| **SON** | SJA-W | 4 | 0 | 4 | - | - |
| **SON** | SJP-W | 2 | 0 | 2 | - | - |
| **SON** | TEM-C | 2 | 0 | 2 | - | - |
| **CPA** | ELO-W | 6 | 0 | 6 | - | - |
| **CPA** | HUJ-W | 4 | 0 | 4 | - | - |
| **CPA** | HUJ-C | 2 | 0 | 2 | - | - |
| **CPA** | LIB-C | 2 | 0 | 1 | - | 1 |
| **CPA** | PEL-W | 4 | 0 | 4 | - | - |
| **CPA** | PLC-W | 2 | 1 | 1^b^ | - | 1 |
| **AZP** | BER-W | 6 | 2 | 6^c^ | - | - |
| **AZP** | CER-W | 6 | 3 | 6^d^ | - | - |
| **AZP** | CER-C | 2 | 0 | 2 | - | - |
| **AZP** | TUL-W | 4 | 1 | 4^e^ | - | - |
| **SMO** | PVE-C | 5 | 1 | - | - | 5^f^ |
| **SMO** | TLA-W | 4 | 0 | 2 | - | 2 |
| **SMO** | TLA-C | 8 | 3 | - | 1^g^ | 7^h^ |
| **SMO** | XIL-W | 2 | 0 | - | - | 2 |
| **CPS** | HUA-W | 4 | 0 | 4 | - | - |
| **CPS** | HUA-C | 2 | 0 | 2 | - | - |
| **YUC** | DZI-W | 10 | 0 | 9 | - | 1 |
| **YUC** | CHO-C | 2 | 1 | - | - | 2^i^ |
| **SON** |  | 22 | 0 | 22 | - | - |
| **CPA** |  | 20 | 1 | 18 | - | 2 |
| **AZP** |  | 18 | 6 | 18 | - | - |
| **SMO** |  | 19 | 4 | 2 | 1 | 16 |
| **CPS** |  | 6 | 0 | 6 | - | - |
| **YUC** |  | 12 | 1 | 9 | - | 2 |
| **W** |  | 70 | 7 | 64 | - | 6 |
| **C** |  | 27 | 5 | 11 | 1 | 15 |
| **Total** |  | **97** | **12** | **75** | **1** | **21** |

^a)^ N plant: number of plants analysed by population; N hetero.: number of heterozygous plants at the *pvr2* locus; N s/s: number of homozygous plants for susceptible alleles (susceptible plants); N R/s: number of heterozygous plants for susceptible and resistant alleles (susceptible plants); N R/R: number of homozygous plants for resistant alleles (resistant plants). ^b)^ 1 heterozygous plant at the *pvr2* locus *pvr2*^+^/*pvr1*^+^; ^c)^ 2 plants *pvr2^2^*^4^/*pvr2^2^*^5^; ^d)^ 3 plants *pvr2^2^*^4^/*pvr2^2^*^5^; ^e)^ 1 plant *pvr2*^+^/*pvr2^2^*^4^; ^f)^ 1 plant *pvr2*^1^/*pvr2*^4^; ^g)^ 1 plant *pvr2*^+^/*pvr2*^1^; ^h)^ 2 plants *pvr2*^1^/*pvr2*^9^; ^i)^ 1 plant *pvr2*^2^/*pvr2*^4^.
