# Supplementary figures and images for: Human Management of a Wild Plant Modulates the Evolutionary Dynamics of a Gene Determining Recessive Resistance to Virus Infection
Source: PLoS Genet. 2016 Aug 4;12(8):e1006214. doi: 10.1371/journal.pgen.1006214 (PMC4973933; doi:10.1371/journal.pgen.1006214)

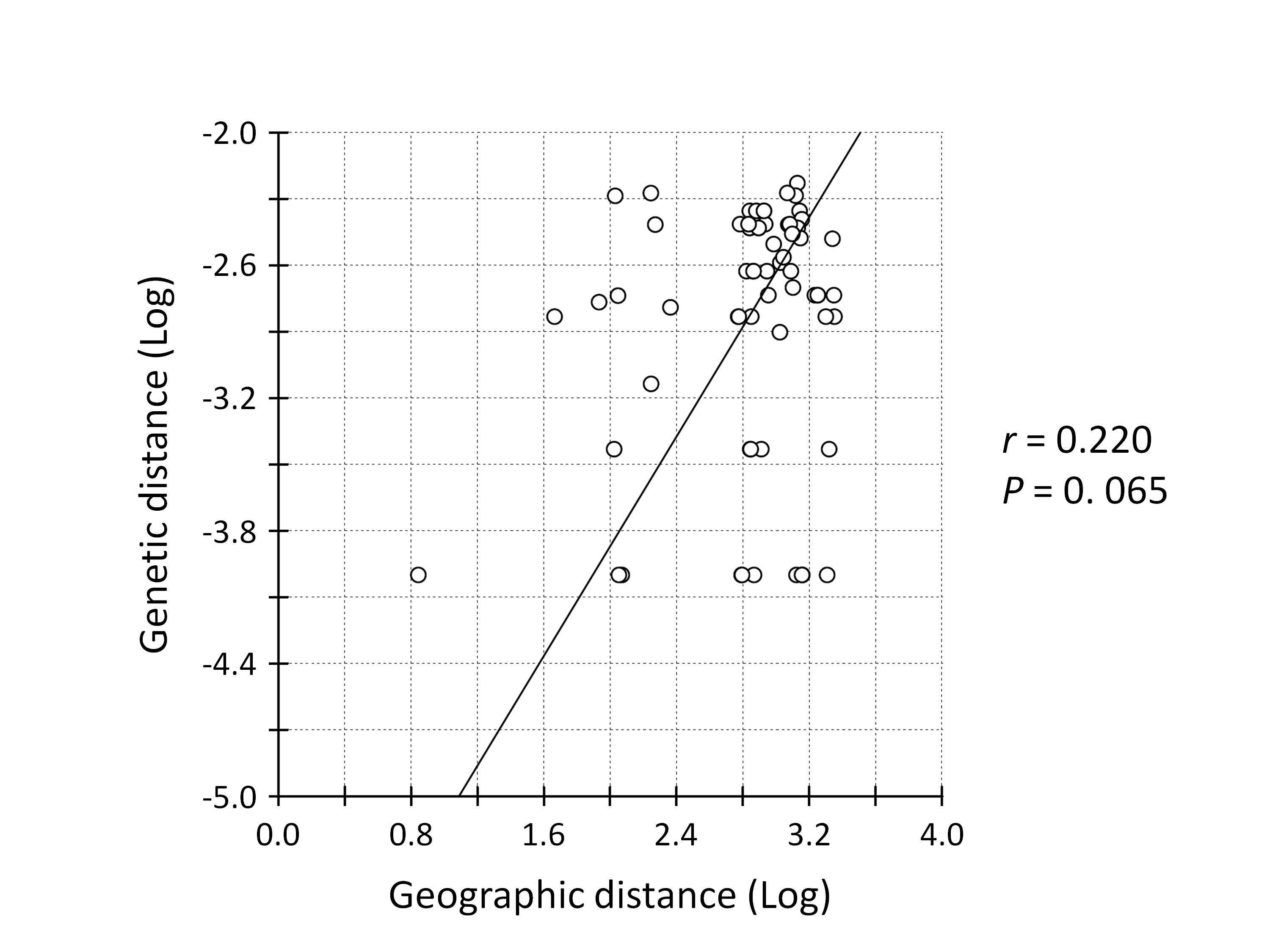

Supplement: S1 Fig — Log-transformed data are presented. (TIF) [file pgen.1006214.s006.tif]

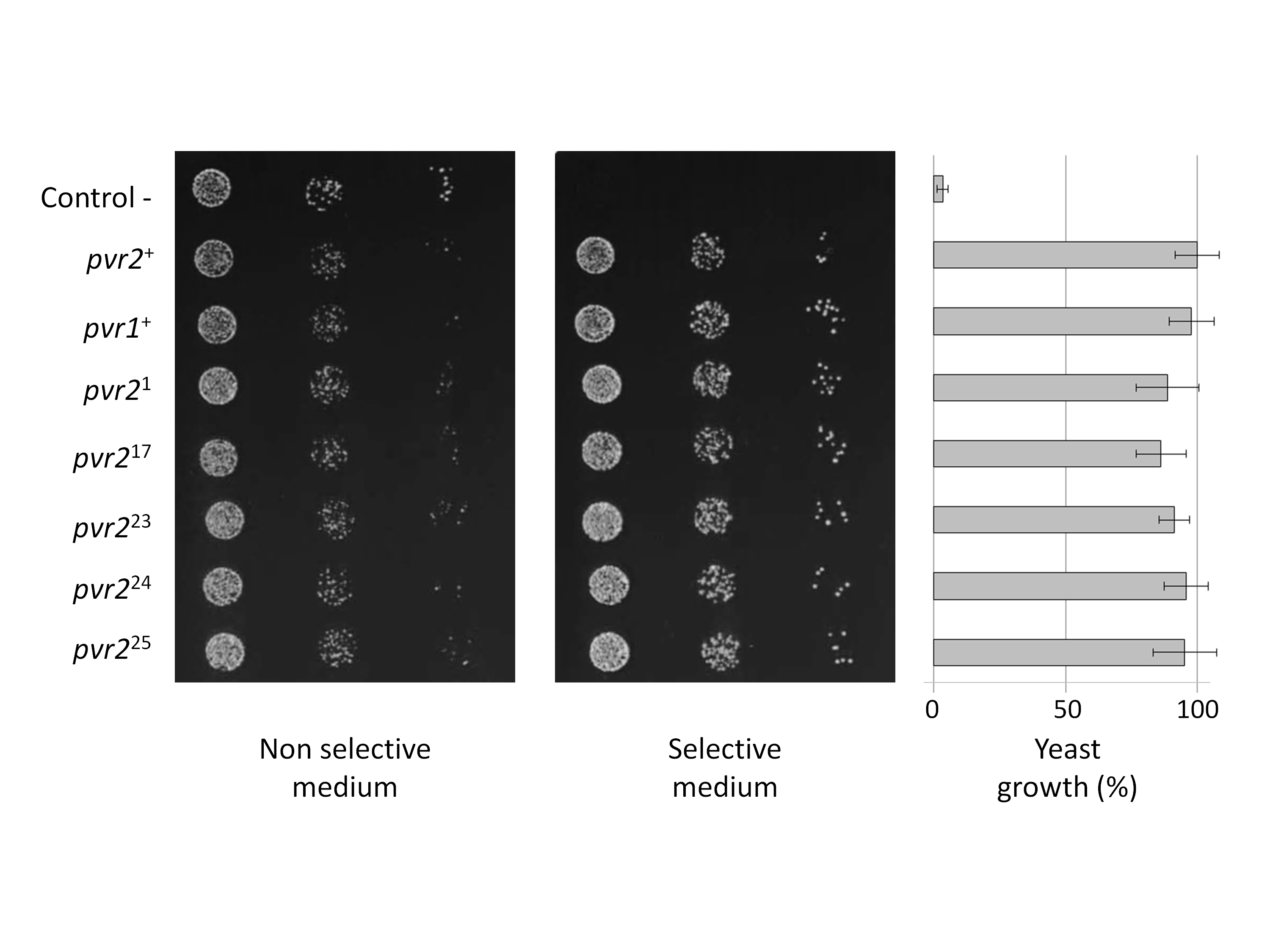

Supplement: S2 Fig — Control -: negative control corresponding to yeast colonies transformed with empty p424GBP/TRP1 plasmids. (TIF) [file pgen.1006214.s007.tif]

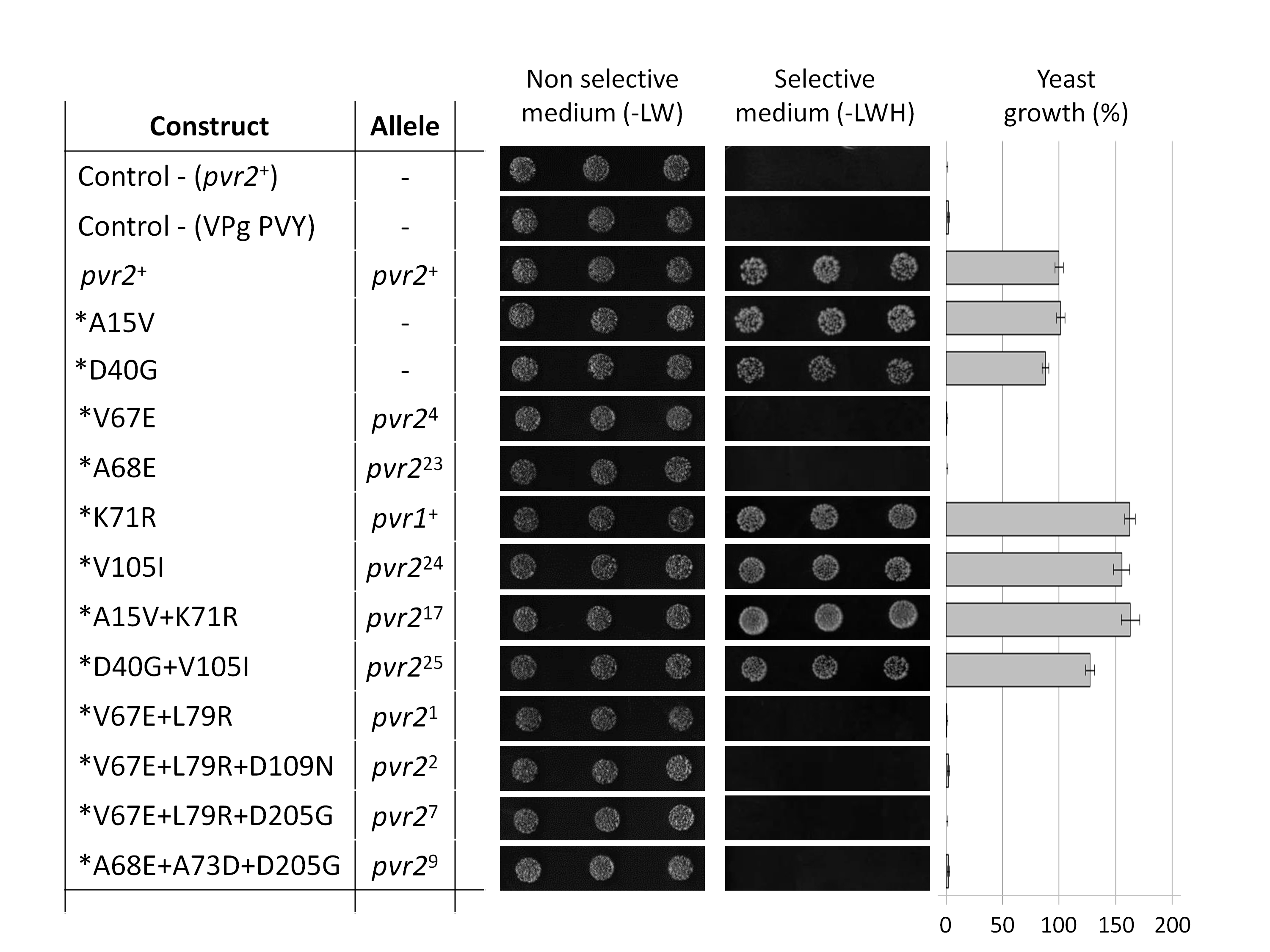

Supplement: S3 Fig — Yeast growth was evaluated by yeast two hybrid assays and was expressed by percentage of the yeast growth on the selective medium (-LWH) compared to the reference yeast colonies co-transformed with pGADT7::pvr2+ and pGBKT7::VPg-PVY; standard errors were obtained after 3 replications of the yeast two-hybrid assays in which 3 independent colonies of each pvr2/eIF4E1-VPg:PVY combination were randomly selected. (TIF) [file pgen.1006214.s008.tif]

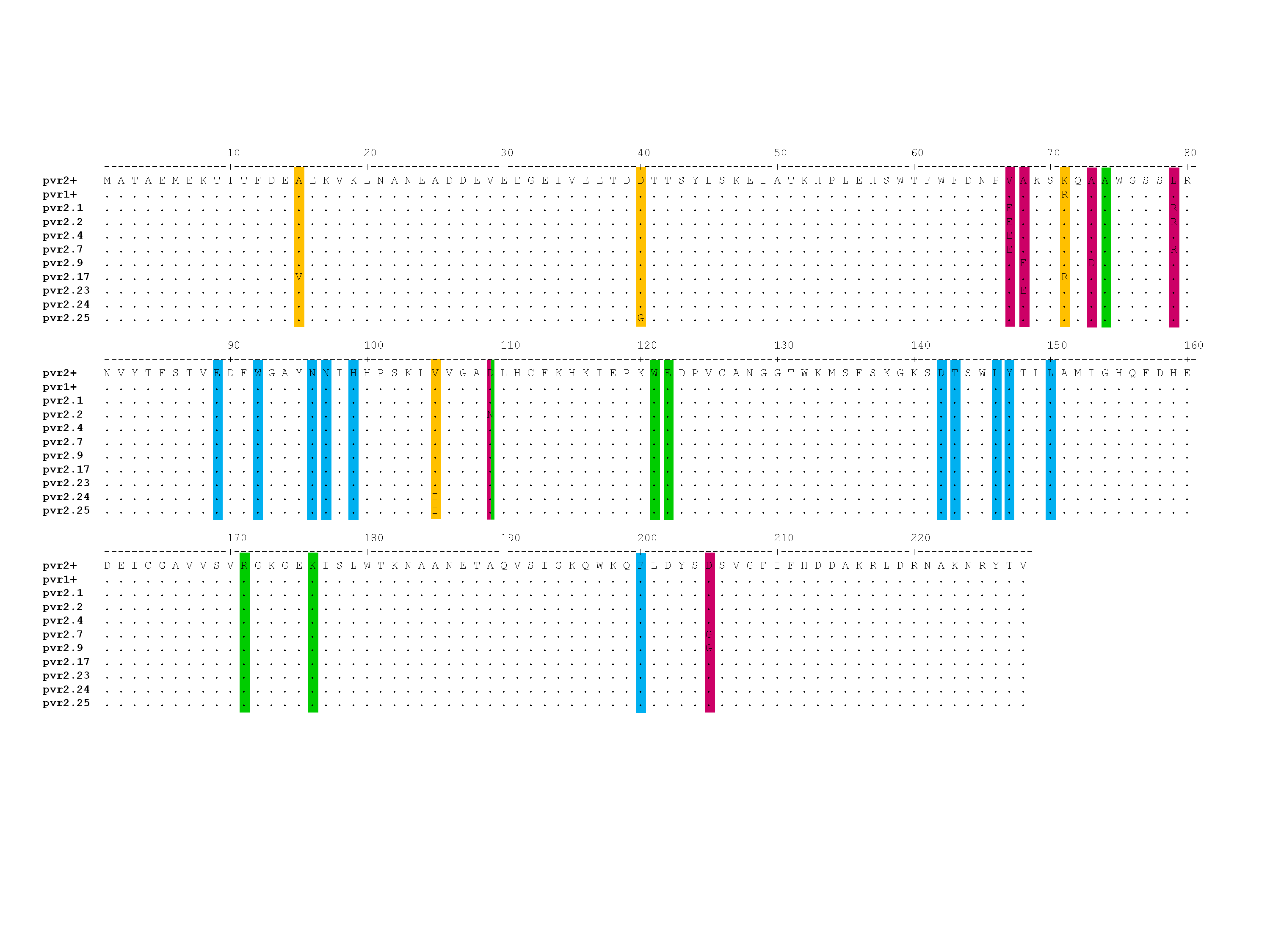

Supplement: S4 Fig — (TIF) [file pgen.1006214.s009.tif]

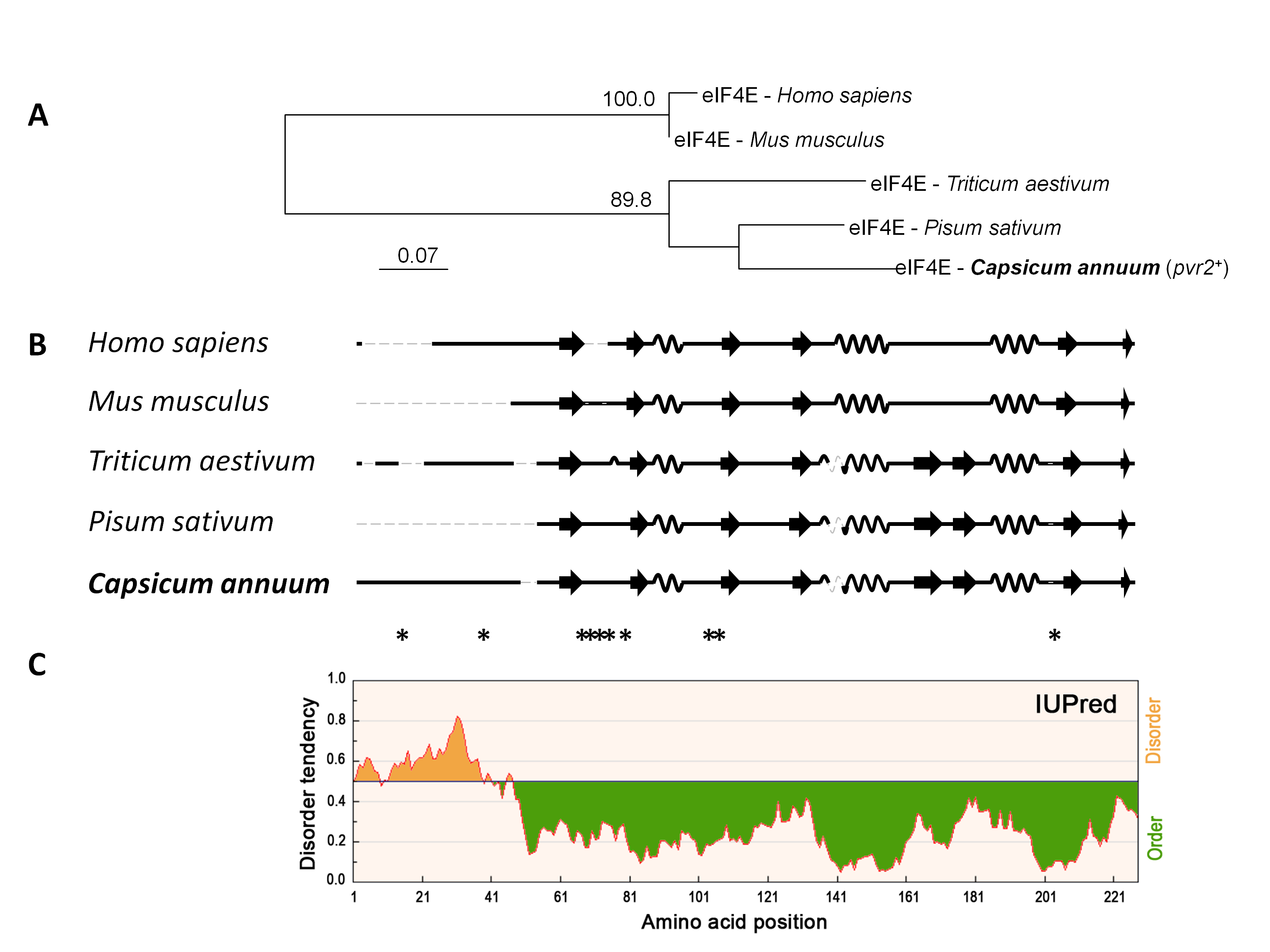

Supplement: S5 Fig — *: localisation of the polymorphic positions identified in the pvr2/eIF4E1 protein of chiltepins. (TIF) [file pgen.1006214.s010.tif]

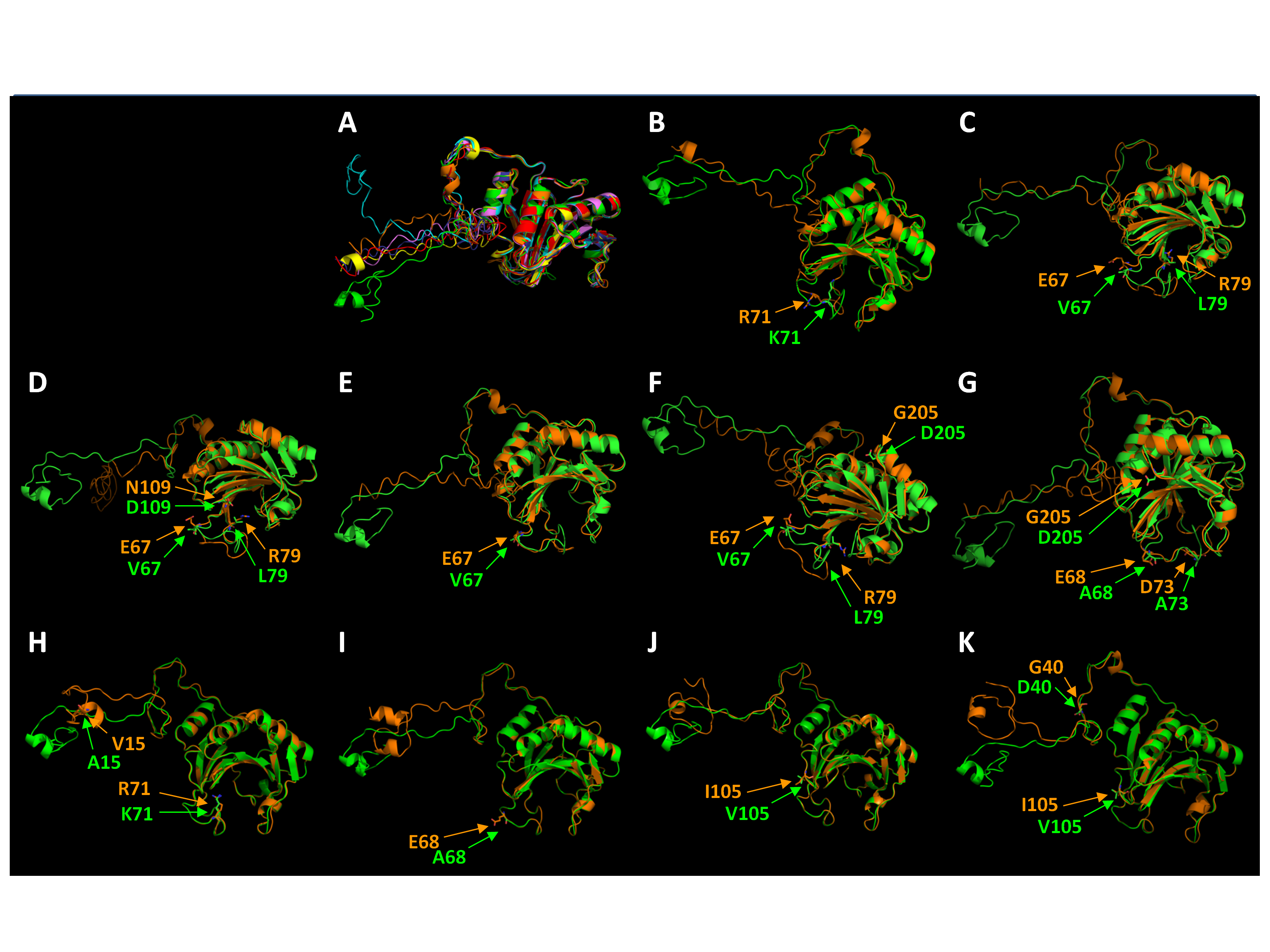

Supplement: S6 Fig — (A) superposition of the models; (B) pvr2+ (green) and pvr1+ (orange), superposition: RMSDbackbone = 1.050 Å; (C) pvr2+ (green) and pvr21 (orange), superposition: RMSDbackbone = 1.107 Å; (D) pvr2+ (green) and pvr22 (orange), superposition: RMSDbackbone = 0.978 Å; (E) pvr2+ (green) and pvr24 (orange), superposition: RMSDbackbone = 1.090 Å; (F) pvr2+ (green) and pvr27 (orange), superposition: RMSDbackbone = 0.999 Å; (G) pvr2+ (green) and pvr29 (orange), superposition: RMSDbackbone = 0.999 Å; (H) pvr2+ (green) and pvr217 (orange), superposition: RMSDbackbone = 0.602 Å; (I) pvr2+ (green) and pvr223 (orange), superposition: RMSDbackbone = 0.633 Å; (J) pvr2+ (green) and pvr224 (orange), superposition: RMSDbackbone = 0.692 Å; (K) pvr2+ (green) and pvr225 (orange), superposition: RMSDbackbone = 0.716 Å. (TIF) [file pgen.1006214.s011.tif]

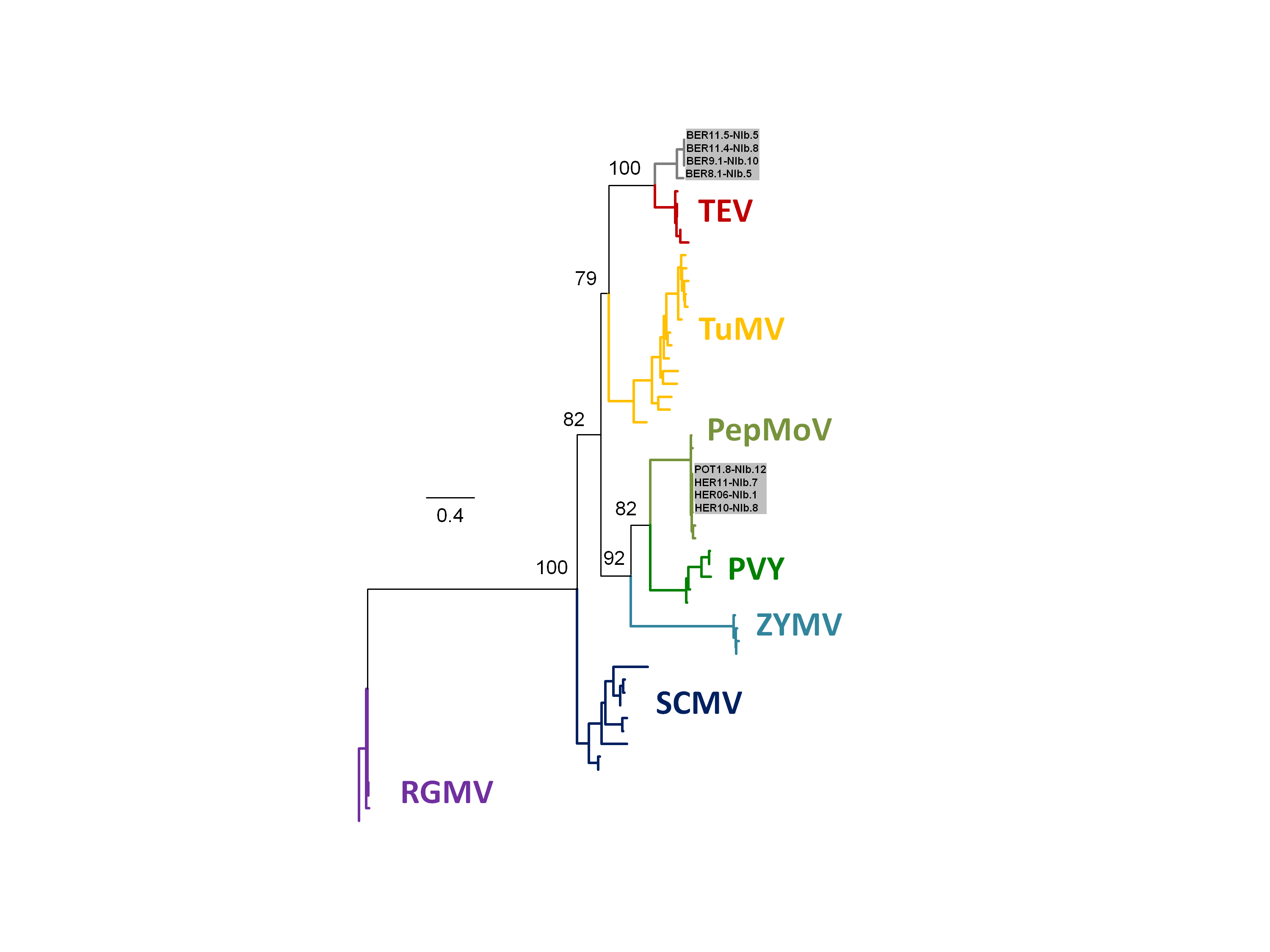

Supplement: S7 Fig — The presented phylogeny was reconstructed based on partial NIb (304nt) by the NJ method, bootstrap values (1000 replicates) are indicated on the nodes. Grey boxes mentioned the potyvirus sequences identified in chiltepin populations. (TIF) [file pgen.1006214.s012.tif]

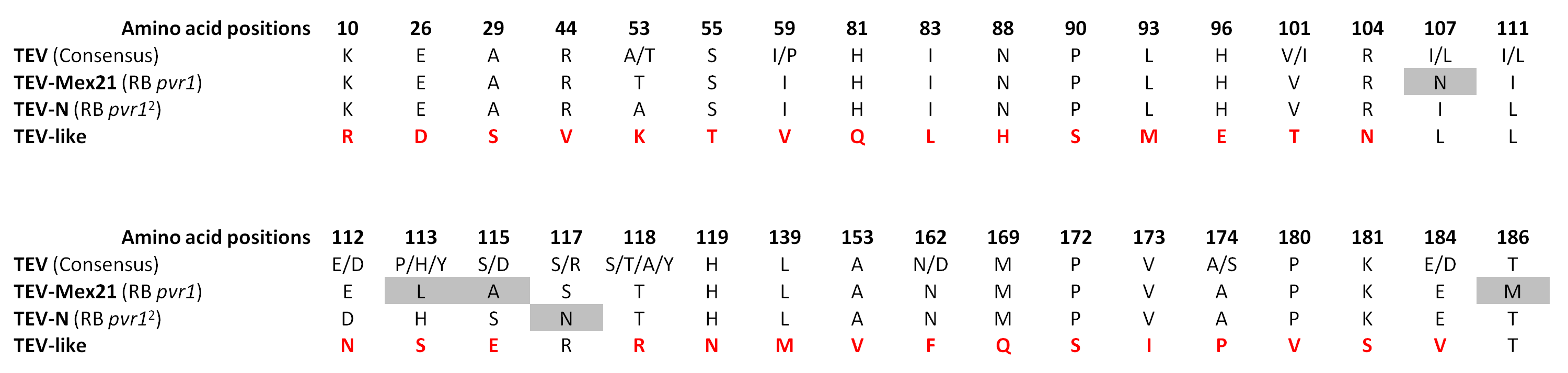

Supplement: S8 Fig — TEV (Consensus): Consensus sequence obtained with 20 world-wide TEV isolates (accession numbers: L38714, M11458, M15239.1, NC_001555.1, DQ986288.1, EF470242.2, JN711120.1, EU334794.1, EU334793.1, EU334792.1, EU334791.1, EU334790.1, 334789.1, EU334788.1, EU334787.1, EU334786.1, EU334785.1, EU334784.1, EU334783.1, JX512812.1); TEV-Mex21 (RB pvr1): Sequence of a pvr1 resistance-breaking TEV isolate (KM282188); TEV-N (RB pvr12): Sequence of a pvr12 resistance-breaking TEV isolate (KM282189); Grey boxes: Mutations putatively involved in the resistance-breaking process; Red letters: discriminating position between TEV-like and TEV. (TIF) [file pgen.1006214.s013.tif]

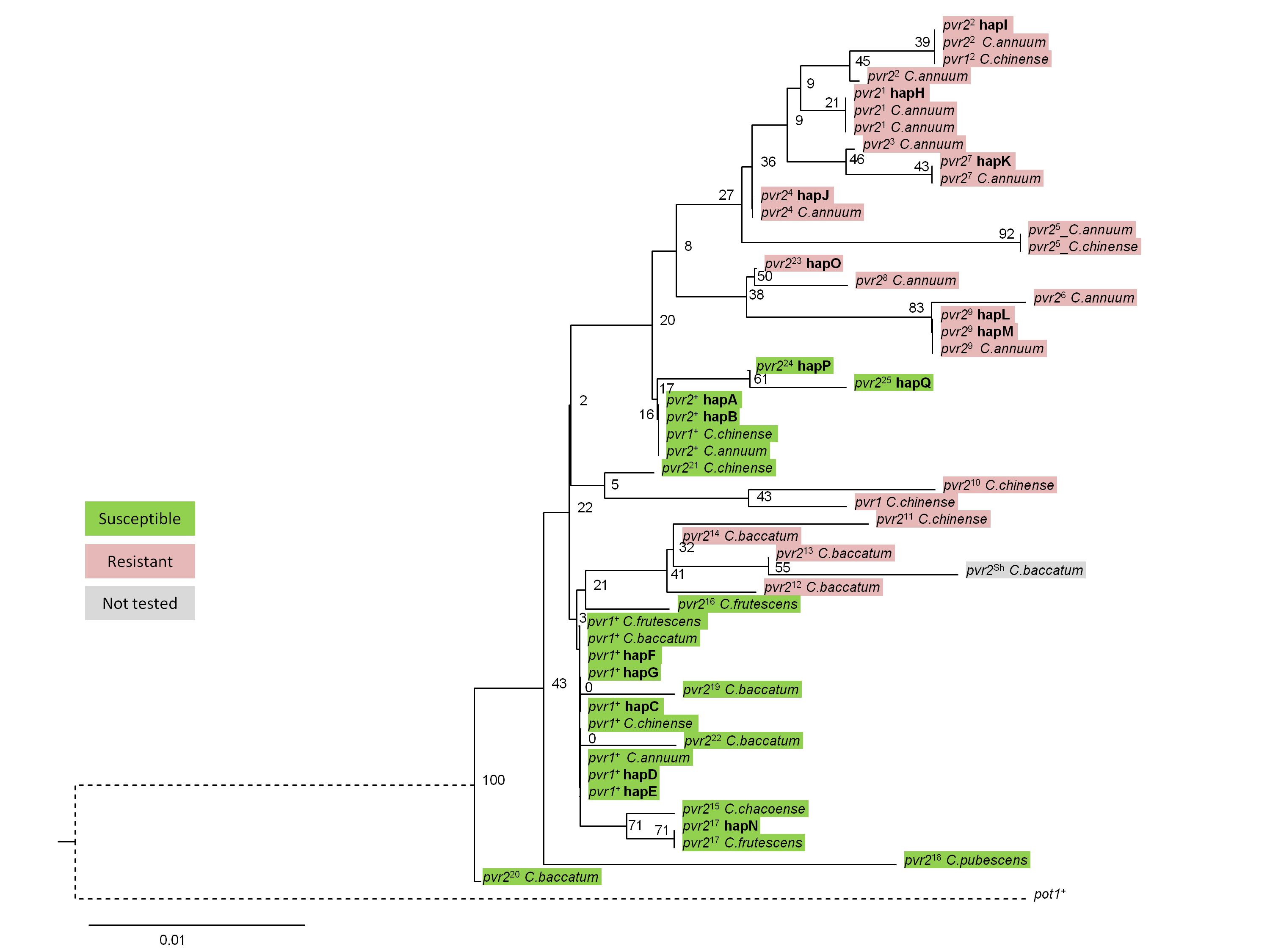

Supplement: S9 Fig — The presented phylogeny was reconstructed by the NJ method, bootstrap values (1000 replicates) are indicated on the nodes. hapA to hapQ are pvr2/eIF4E1 haplotypes identified in chiltepin populations; pvr2+ and pvr21 to pvr29 are pvr2/eIF4E1 haplotypes described in [23]; pvr1+, pvr1 and pvr12 are described in [22]; pvr210 to pvr222 are described in [45]; pot1+: Potyvirus susceptibility allele from tomato (accession number AY723733). Green label: susceptible allele; red label: resistant allele; grey label: not characterized allele. (TIF) [file pgen.1006214.s014.tif]

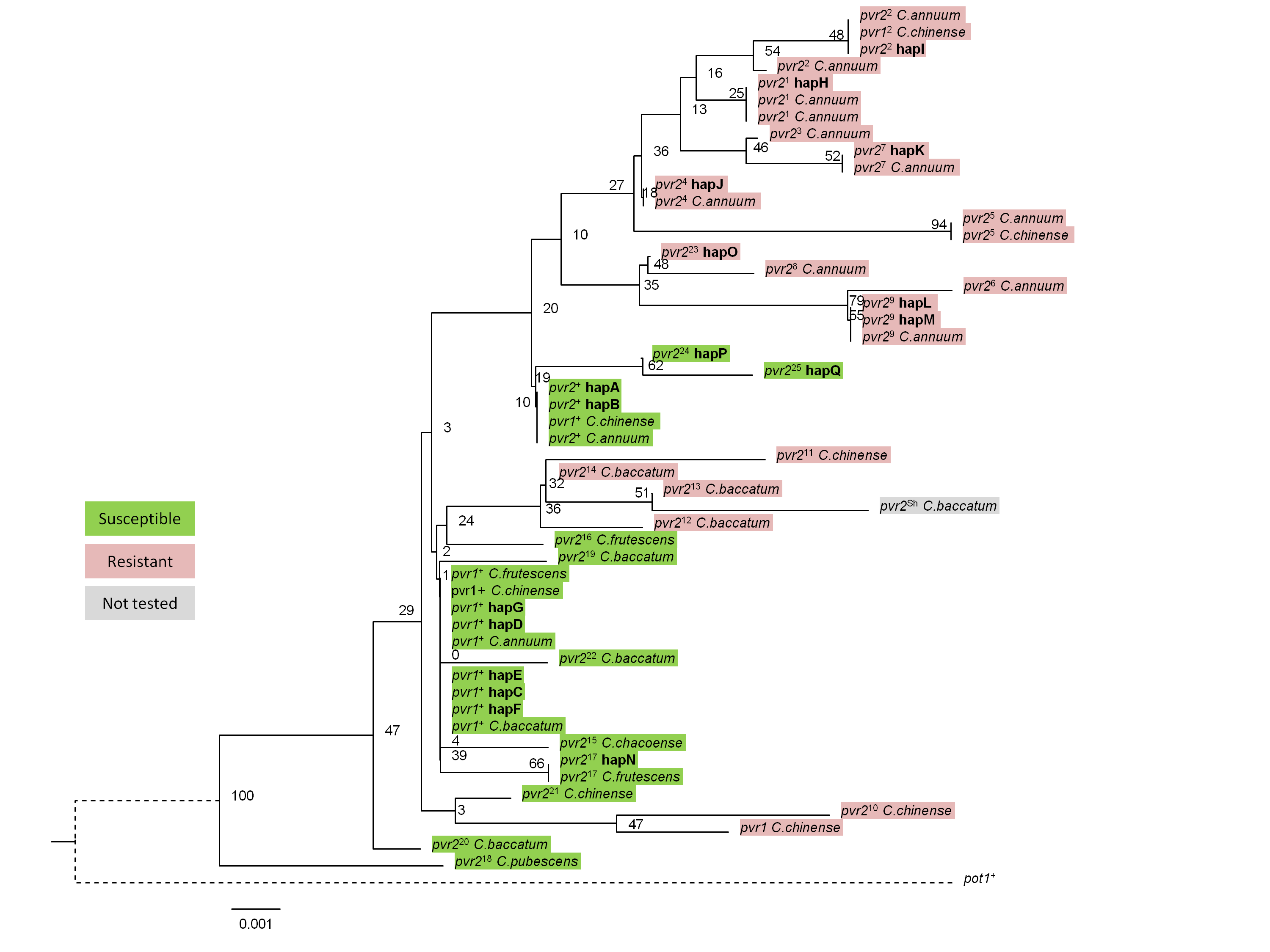

Supplement: S10 Fig — The presented phylogeny was reconstructed by the NJ method, bootstrap values (1000 replicates) are indicated on the nodes. hapA to hapQ are pvr2/eIF4E1 haplotypes identified in chiltepin populations; pvr2+ and pvr21 to pvr29 are pvr2/eIF4E1 haplotypes described in [23]; pvr1+, pvr1 and pvr12 are described in [22]; pvr210 to pvr222 are described in [45]; pot1+: Potyvirus susceptibility allele from tomato (accession number AY723733). Green label: susceptible allele; red label: resistant allele; grey label: not characterized allele. (TIF) [file pgen.1006214.s015.tif]

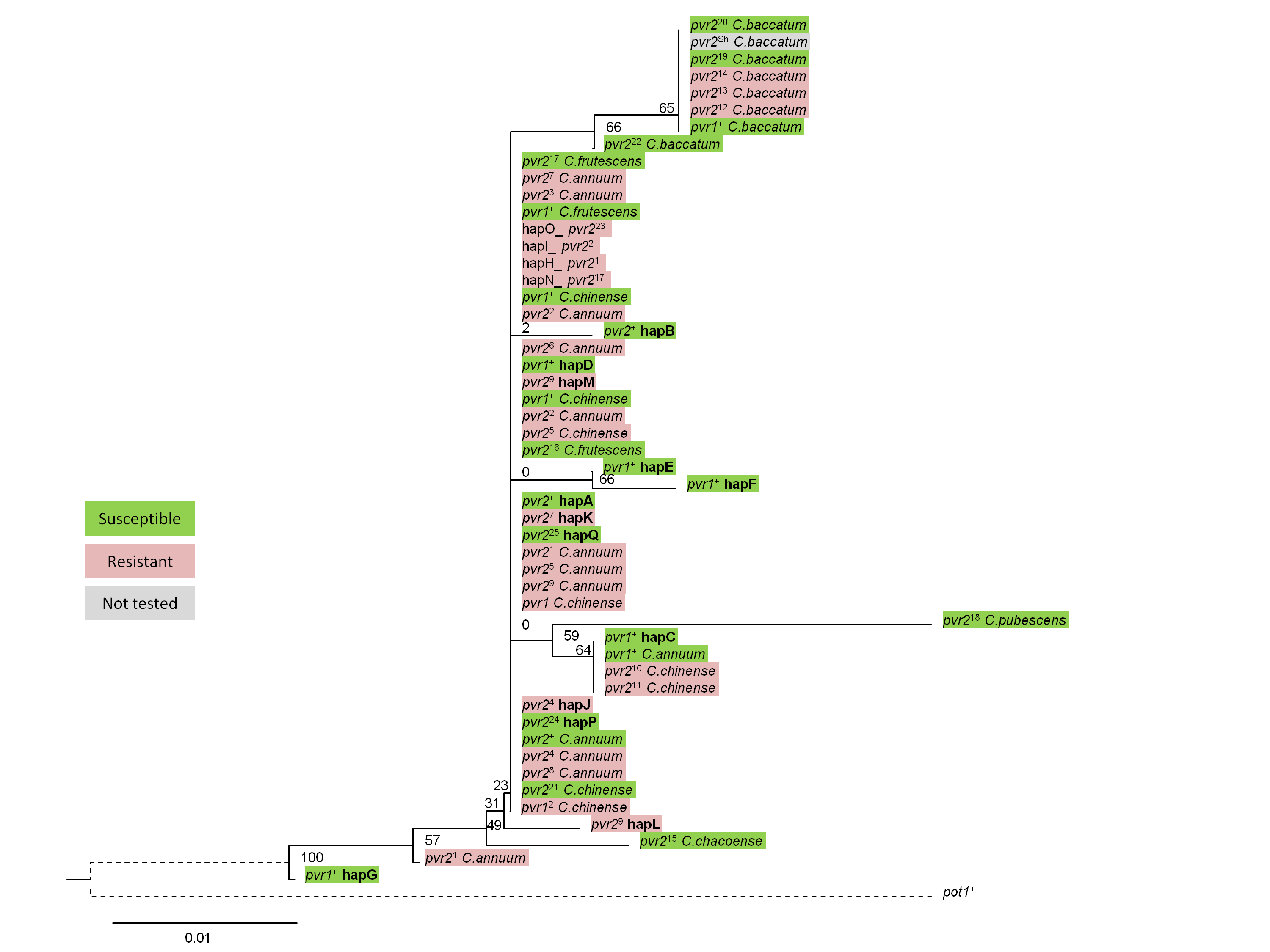

Supplement: S11 Fig — The presented phylogeny was reconstructed by the NJ method, bootstrap values (1000 replicates) are indicated on the nodes. hapA to hapQ are pvr2/eIF4E1 haplotypes identified in chiltepin populations; pvr2+ and pvr21 to pvr29 are pvr2/eIF4E1 haplotypes described in [23]; pvr1+, pvr1 and pvr12 are described in [22]; pvr210 to pvr222 are described in [45]; pot1+: Potyvirus susceptibility allele from tomato (accession number AY723733). Green label: susceptible allele; red label: resistant allele; grey label: not characterized allele. (TIF) [file pgen.1006214.s016.tif]
